# Gut microbiota composition in responders vs. non-responders with hepatocellular carcinoma under ICI therapy: a systematic review and meta-analysis

**DOI:** 10.3389/fmed.2026.1928103

**Published:** 2026-09-09

**Authors:** Guo Lin, Bowei Huang, Shun Chen, Hangbin Zheng, Wenxin Huang, Juan Hu, Liangyi Zhou, Liwu Chen

**Affiliations:** 1 The Second Affiliated Hospital of Fujian University of Traditional Chinese Medicine, Fuzhou, Fujian, China; 2 The First Clinical Medical College, Fujian University of Traditional Chinese Medicine, Fuzhou, Fujian, China

**Keywords:** gut microbiome, hepatocellular carcinoma, immune checkpoint inhibitors, metabolites, prognosis

## Abstract

**Introduction:**

The associations between gut microbiota and disease progression, as well as the prognostic implications of gut microbiota, remain unclear in patients with hepatocellular carcinoma (HCC) treated with immune checkpoint inhibitors (ICIs). This study aimed to delineate gut microbiome differences among ICI-treated patients with intermediate-to-advanced HCC and assess their correlations with clinical outcomes via meta-analysis.

**Methods:**

We searched 4 databases (PubMed, Embase, Web of Science, and the Cochrane Library) and collected related articles published before May 31, 2026. We calculated standard mean differences (SMDs) and 95% confidence intervals (CIs) using a random-effects model. Summarized hazard ratios (HRs) with 95% CIs were used to assess gut microbiome associations with overall survival (OS) and progression-free survival (PFS). Subgroup, sensitivity, and publication bias analyses were further conducted, with all statistical analyses performed using RevMan and R.

**Results:**

A total of 13 eligible papers were included. (1) The meta-analysis of Alpha diversity indices between R and NR was no significant difference, but over 68% of the studies reported significant differences in beta diversity. (2) At the phylum level, *Actinobacteriota* were significantly enriched in the R group. At the genus level, depleted *Bacteroides* exhibited distinct relative abundance in the NR group. (3) Protective taxa (*Collinsella*, *Ruminococcus.AF25_28AC*, *Ruminococcus.AM42_11*, *Erysipelotrichaceae bacterium-GAM147*, *Bacteroides stercoris/Parabacteroides merdae*) correlated with reduced mortality risk. Risky taxa (*Bacteroides.AF20_13LB*, *Veillonella.atypica*, *Veillonella.AF13_2*, *Veillonellaceae* and *s_Actinomyces_sp_ICM47*) showed worse OS outcomes. (4) PFS-protective taxa (*Collinsella*, *Bacteroidales zoogleoformans*, *Ruminococcus.AF25_28AC*, *Ru-minococcus callidus*, and *Erysipelotrichaceae bacterium GAM147*) correlated with reduced progression risk. PFS-detrimental taxa (*Prevotella 9*, *Bacteroides_AF20_13LB*, *Veillonella atypica*, and *Lachnoclostridium*) showed worse outcomes. (5) Responders showed upregulated bile acid metabolism, fatty acid biosynthesis, methanogenesis and carbon fixation pathways associated with beneficial genera, while non-responders exhibited elevated secondary bile acid metabolism, arginine biosynthesis and carbohydrate transport pathways linked to detrimental taxa.

**Conclusions:**

HCC patients with varied treatment responses exhibit characteristic shifts in gut flora. Compositional and functional variations of intestinal microbes serve as potential indicators of disease status, promising prognostic predictors, and references for clinical therapeutic decisions.

**Systematic Review Registration:**

https://www.crd.york.ac.uk/prospero/, PROSPERO CRD420261363281.

## Introduction

Hepatocellular carcinoma is a malignant tumor with high morbidity and mortality worldwide. A large proportion of patients with intermediate and advanced HCC are ineligible for surgical resection (1). In recent years, immune checkpoint inhibitors (ICIs), transarterial chemoembolization (TACE) and targeted therapy have become the mainstay treatments for these patients, which are expected to improve the response rate and survival of individuals with advanced HCC (2, 3). However, patient responses to ICI therapy vary substantially, and not all advanced HCC patients can benefit from this treatment (4). Despite the expanding clinical scope and growing utilization of ICIs, reliable pretreatment biomarkers to precisely predict ICIs’ therapeutic response in HCC patients are still lacking (5). Given the dynamic changes in the tumor microenvironment and the uncertainty regarding disease progression and optimal timing for treatment adjustment, it is of great clinical significance to identify biomarkers that can predict treatment efficacy and long-term survival in HCC patients receiving ICI therapy.

The gut-liver axis is a vital physiological pathway connecting the intestine and liver, and its dysfunction is closely linked to the occurrence and progression of HCC (6). As a core component of the gut-liver axis, the gut microbiota plays a pivotal role in maintaining intestinal homeostasis and regulating hepatic inflammation and immune function (7).

Accumulating evidence reveals prominent gut microbiota dysbiosis in patients with HCC, characterized by shifts in microbial diversity and aberrant abundance of certain bacterial phyla and genera (8–10). Nevertheless, existing studies merely reveal correlations between gut microbiota and intermediate/advanced HCC. Most of them lack comparative analysis of treatment responses, making it difficult to clarify the causal relationship and multivariate interactions between gut microbiota and therapeutic outcomes. A core clinical challenge lies in determining the optimal management strategy following progression with ICI-based therapies (11). For patients who fail to achieve therapeutic response to first-line regimens, further investigations are warranted to develop interventions capable of converting non-response to response and improving clinical outcomes.

Accordingly, this study conducted a systematic review and meta-analysis to synthesize available evidence on gut microbiota composition in patients with intermediate and advanced HCC. We aimed to characterize the overall profiles of gut microbiota in this patient population and further compare microbial features between treatment responders and non-responders, as well as explore the impacts of ICI therapy on gut microbiota. The findings will provide high-level evidence for the clinical application of gut-liver axis regulation in the management of intermediate and advanced HCC.

## Materials and methods

This study was performed in accordance with the Preferred Reporting Items for Systematic Reviews and Meta-Analysis (PRISMA) statement (12), and was registered at the National Institute for Health Research PROSPERO (registration number: CRD420261363281).

### Search strategy

The literature was limited to English studies published before May 31, 2026. Two authors independently performed the literature search in PubMed, the Cochrane Library, Embase, and Web of Science. The search strategy combined the following keywords via Boolean operators AND/OR: “gut microbiome”, “microbiota”, “microbiome”, “bacteria”, “microbial”, “flora”, “microbe”, “cirrhosis”, “hepatocellular carcinoma”, and “Immune checkpoint inhibitor” (Supplementary Table S1).

### Inclusion and exclusion criteria

According to Response Evaluation Criteria in Solid Tumors (RECIST/mRECIST), patients with complete response (CR), partial response (PR), or stable disease (SD) were defined as the responder group (R), whereas patients with stable disease lasting less than 6 months or progressive disease (PD) were defined as the non-responder group (NR).

The inclusion criteria were as follows: adult patients with histologically—or radiologically-confirmed hepatocellular carcinoma (HCC) receiving immune checkpoint inhibitor (ICI)-based therapy; observational cohort/correlative human studies; fecal microbiota profiling via 16S rRNA amplicon or whole-genome shotgun metagenomic sequencing, with extractable microbiota data (alpha-/beta-diversity, phylum-to-species-level taxonomic abundance); patient stratification into ICI-responders (R) and non-responders (NR) by RECIST criteria and reporting of at least one outcome (microbial profiles, overall survival, or progression-free survival); and full-text English articles published before May 31, 2026.

Meanwhile, we excluded duplicate studies, case reports, commentaries, protocols, meta-analyses, review articles, animal experiments and studies detecting microbiota only in non-fecal specimens (tissue, blood, oral swabs).

### Data extraction and quality assessment

Two authors extracted data from the final included studies independently, such as first author, publication year, publication type, country, patient characteristics, age of patients, numbers of patients and controls, follow-up duration, treatment methods, survival analysis, microbiota detection methods, and quality score. We extracted data on alpha diversity indices, beta diversity and statistical significance results, and relative abundances of bacterial taxa at phylum, family, and genus levels from each eligible study. WebPlot Digitizer (v4.5) (13) was utilized to extract graphical data when original numerical values were unavailable. The Newcastle–Ottawa Scale (NOS) was adopted to assess the quality of included studies, with total scores spanning 0–9; higher scores indicate better methodological quality (14). For ambiguous parts, a third evaluator was required to participate in the discussion and make the final decision. Notably, the Newcastle-Ottawa Scale (NOS) was used for post-inclusion risk-of-bias assessment rather than as an exclusion criterion; lower-quality studies were retained and assessed in sensitivity analyses.

### Heterogeneity assessment

We pre-specified potential sources of between-study heterogeneity for qualitative assessment, including: (1) patient demographic characteristics (age distribution, geographic region, ethnicity); (2) HCC etiology (viral hepatitis B/C, alcohol-related, metabolic-associated steatohepatitis-related, cryptogenic cirrhosis); (3) disease stage (unresectable HCC, advanced HCC; prior TACE/local ablation history); (4) microbiota detection platforms (16S rRNA sequencing with different variable regions, metagenomic shotgun sequencing); (5) fecal sample handling protocols (storage condition); (6) ICI regimens (combination therapy, drug agents). These variables were extracted and summarized to interpret observed statistical heterogeneity (*I*^2^ and Cochran's *Q*).

### Summary outcome measures and statistical analysis

Microbiota abundance in each group was summarized by mean ± standard deviation (SD). Pooled effects were calculated as standardized mean differences (SMD) with 95% confidence intervals (CIs). We also assessed heterogeneity using Cochran's *Q* and *I*^2^ statistics. The summary hazard ratio (HR) with 95% CI was derived from each study, with a preference for HR data. From included studies, we retrieved *β*-diversity metrics and documented taxonomic shifts (enriched or depleted), classifying each taxon as increased, decreased, or not assessed. These categorized outcomes were presented via a heatmap for clear visual comparison. We qualitatively summarized metabolic pathways and metabolite-microbiota correlation outcomes that were reported in the included primary studies. We performed a sensitivity analysis by omitting one study at a time to evaluate the influence of each study. Potential publication bias was assessed using Trim-and-fill funnel plot and Egger's tests. Statistical analysis was performed using RevMan and R, and statistical significance was set to *P* < 0.05.

## Results

### Characteristics and quality of the included studies

**Figure 1:**
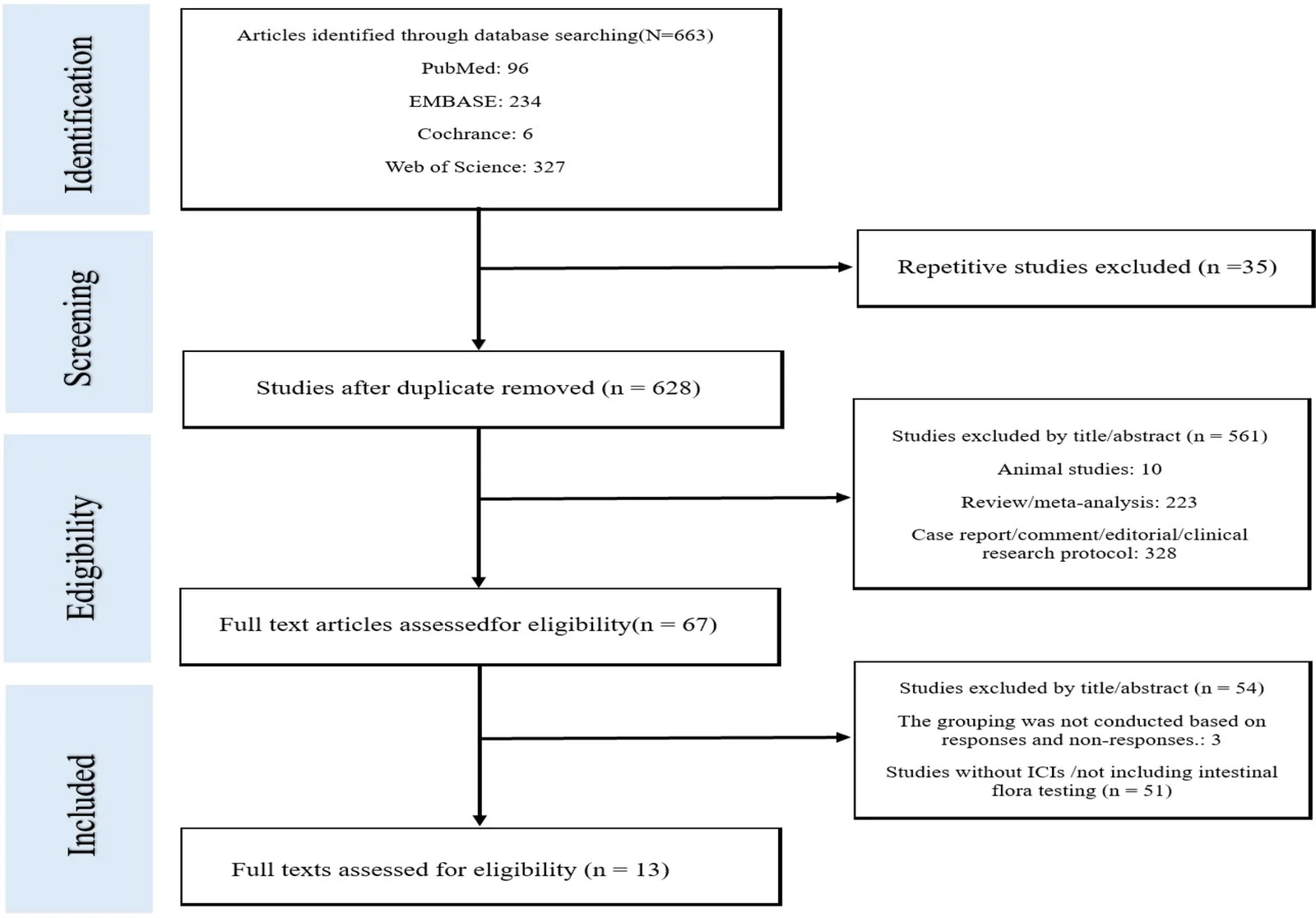
Flow diagram of the literature search.

A total of 663 articles were initially searched in the database, and we selected abstracts and checked the data. Ultimately, 13 articles were analyzed in this study. A detailed explanation of the selection procedure can be found in Figure 1.

All 13 included cohort studies achieved NOS scores ranging from 6 to 9 points, with three studies rated as moderate quality (NOS = 6) and the remainder classified as high quality (NOS ≥ 7). Common deduction items include limited descriptions regarding cohort comparability in study design or analysis and limited follow-up descriptions (Supplementary Table 2). We summarized the characteristics of the included articles, with detailed information presented in Table 1. All of the studies were cohort studies, two of which performed animal experiments to validate their research findings. With respect to the individual studies, the total number of included participants varied from 8 to 107. Most of the studies [*n* = 11 (84.6%)] were conducted in Asian countries, 1(7.7%) in North America and 1 (7.7%) in European countries. Most included HCC patients were at intermediate-to-advanced stages, and HBV infection was the predominant etiology. Half of the included studies used combination targeted therapy, while some patients received local therapies including TACE. The gut microbiota was obtained from fecal samples. One study combined 16S rRNA gene sequencing and whole-genome shotgun (WGS) metagenomic sequencing for microbial profiling. Excluding this study, 16S ribosomal RNA sequencing was the most widely adopted stool sample processing method (*n* = 7). Among these seven studies, five targeted the V3–4 variable region, one targeted the V4 variable region, and one targeted the V1–2 variable region. Metagenomic sequencing was the second most common approach (*n* = 4), followed by whole-genome shotgun sequencing (*n* = 1).

**Table 1:** Characteristics of the included studies and subjects.

Author	Country	Definite of HCC	Study design	Total patients	Group	Population	ICIs	Age	Microbiome assessment method	Survival outcomes	Clinical/metabolic outcomes
Inukai et al. (15)	Japan	uHCC	Single-center cohort study	37	HCC(R/NR)	28/9	Atezolizumab	63–85	16S rRNA gene V3-V4 regions	PFS/OS	NA
Chung et al. (16)	Korea	uHCC	Single-site correlative study	8	HCC(R/NR)	5/3	Nivolumab	58.0–66.25	16S rRNA gene V3-V4 regions	NA	NA
Lee et al. (17)	China	uHCC	Single-center cohort study	93	HCC(R/NR)/CPA/NC	21/22/33/17	Nivolumab/pembrolizumab	58.8–67.3	16S rRNA gene V3-V4 regions	PFS/OS	+
Mao et al. (18)	China	uHCC/BTC	Single-center cohort study	65	HCC + BTC (R/NR)	32/33	Anti-PD-1	NA	Metagenomic sequencing	PFS/OS	+(KEGG)
Ponziani et al. (19)	Italy	HCC	Single-center cohort study	11	HCC(R/NR)	6/5	Tremelimumab/Durvalumab	66–80	16S rRNA gene V3-V4 regions	NA	NA
Xin et al. (20)	China	uHCC	Single-center cohort study	45	HCC(R/NR)	30/15	Zimberelimab/tislelizumab/camrelizumab	NA	Whole-genome shotgun sequencing	PFS/OS	NA
Zheng et al. (21)	China	uHCC	Single-center cohort study	8	HCC(R/NR)	3/5	Camrelizumab	NA	Metagenomic sequencing	NA	+(KEGG)
Zhu et al. (22)	China	uHCC	Single-center cohort study	80	HCC(R/NR)	38/42	Toripalimab/camrelizumab	57	Metagenomic sequencing	PFS/OS	+(KEGG)
Tanaka et al. (23)	Japan	aHCC	Single-center cohort study	29	HCC(R/NR)	15/14	Atezolizumab	66.5–81	16S rRNA gene V1-V2 regions	PFS	NA
Shen et al. (24)	China	aHCC	Single-center cohort study	36	HCC(R/NR)	10/26	Anti-PD-1/anti-PD-L1	23.1–86.6	16S rRNA gene V3-V4 regions/Whole-genome shotgun sequencing	OS	NA
Zhao et al. (25)	China	aHCC	Single-center cohort study + animal experiments	65	HCC(R/NR)	50/15	Anti-PD-1	58.3–68.5	Metagenomic sequencing	PFS/OS	+(KEGG)
Fujii et al. (26)	Japan	aHCC	Single-center cohort study	107	HCC(R/NR) vsCON	11/11/85	Atezolizumab	69–89	16S rRNA gene V3-V4 regions	NA	NA
Varela et al. (27)	USA	early-stage resectable HCC/aHCC	Single-center cohort study + animal experiments	50	HCC(R/NR)	10/40	Nivolumab/pembrolizumab/atezolizumab/pembrolizumab	45–84	16S rRNA gene V4 regions	NA	NA

HCC, hepatocellular carcinoma; uHCC, unresectable HCC; aHCC, advanced HCC; ICIs, immune checkpoint inhibitors; OS, overall survival; PFS, progression-free survival; KEGG, Kyoto encyclopedia of genes and genomes; NA, not announced.

### Gut microbiota diversity in alpha and beta diversity

**Figure 2:**
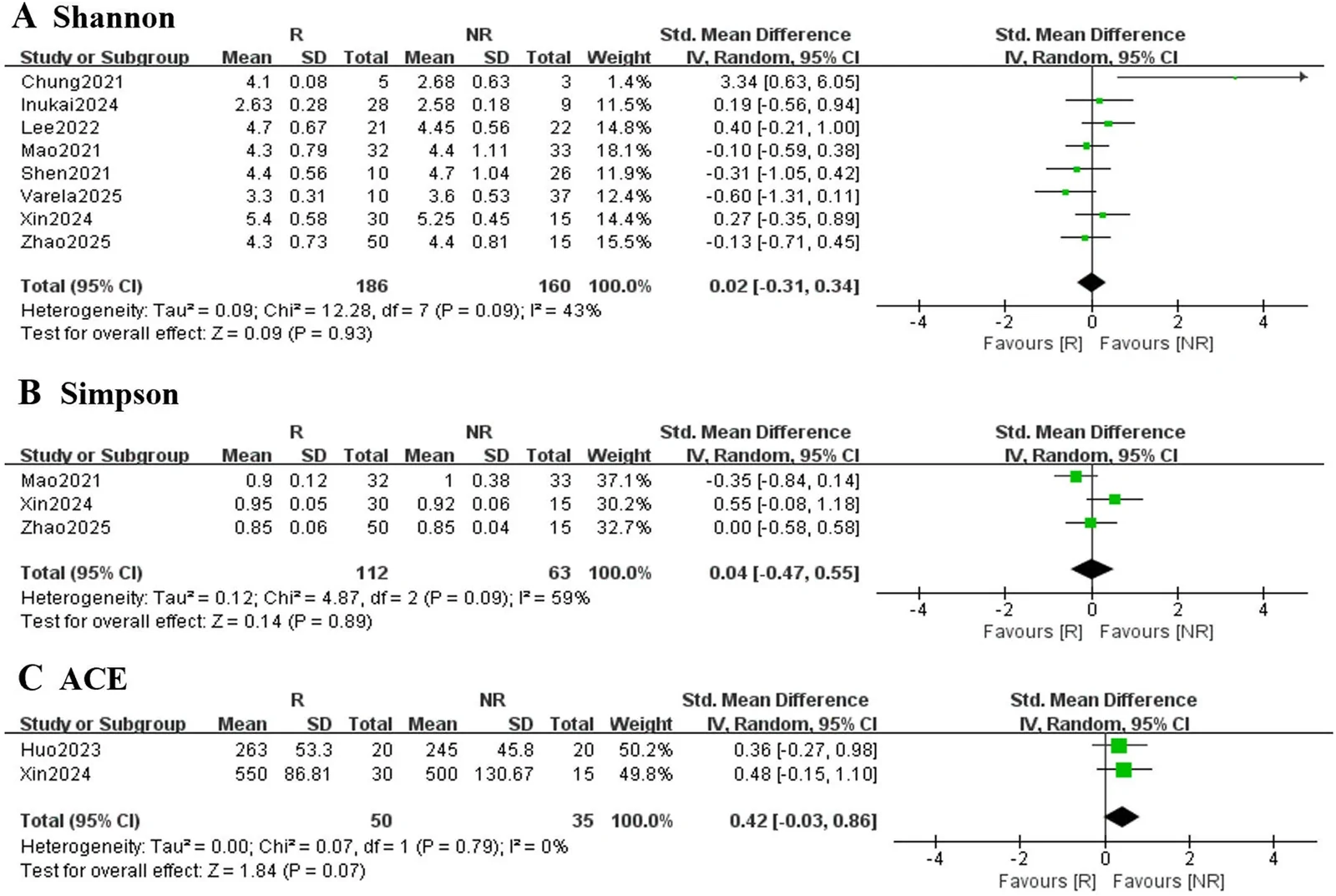
Alpha diversity indices of patients between R and NR in HCC. **(A)** The Shannon index results; **(B)** the Simpson index results; **(C)** ACE index results.

**Figure 3:**
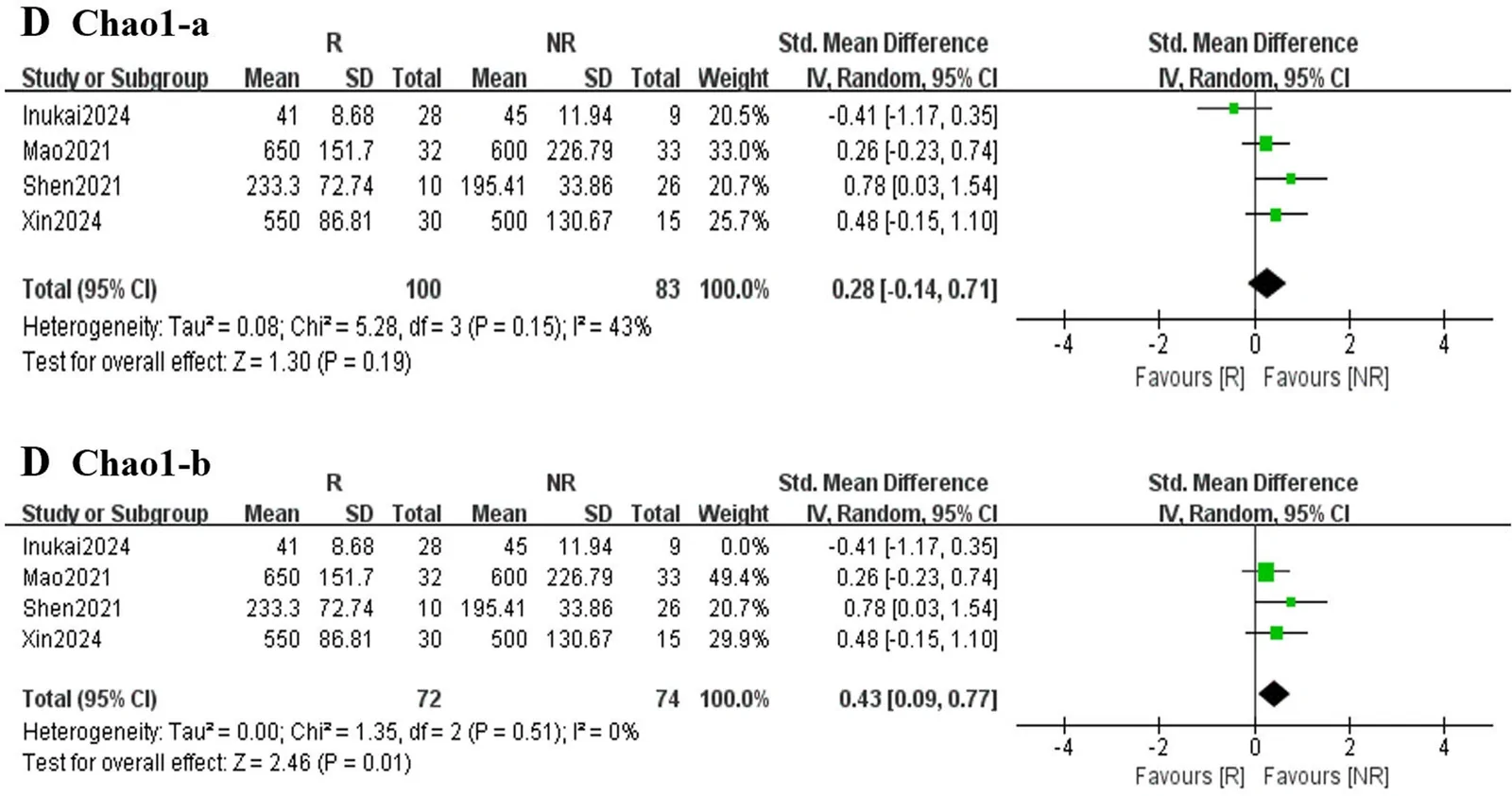
Chao1 index results: **(a)** pooled analysis including all four studies; **(b)** sensitivity analysis after excluding the outlier study (Inukai2024). R, responders; NR, non-responders; SMD, standardized mean difference; 95% CI, 95% confidence interval; *I*^2^, *I*-square heterogeneity statistic.

The alpha diversity of gut microbiota can be quantified using the Shannon, Simpson, Chao 1, ACE, Observed, PD, and Pielou indices. Only one study reported data for the Observed, PD, and Pielou indices respectively; therefore, a meta-analysis could not be performed. In total, 8 studies compared the Shannon index between R and NR in HCC (Figure 2), among which only 1 study detected a significant intergroup difference. Three studies compared the Simpson index, and 2 studies compared the ACE index. Shannon (SMD = 0.02, 95% CI = −0.31 to 0.34, *P* = 0.93), Simpson (SMD = 0.04, 95% CI = −0.47 to 0.55, *P* = 0.89) and ACE (SMD = 0.42, 95% CI = −0.03 to 0.86, *P* = 0.07). Heterogeneity across studies varied for each alpha diversity index, with *I*^2^ values ranging from 0% (ACE) to 59% (Simpson). Therefore, a random-effects model was adopted for the pooled analyses. Four studies compared the Chao1 index (SMD = 0.28, 95% CI = −0.14 to 0.71, *P* = 0.19). There was significant heterogeneity among the studies (*I*^2^ = 43%) as shown in Figure 3. We conducted a sensitivity analysis by omitting one study at a time. These analyses indicated that Inukai2024 (15) contributed the most to the heterogeneity observed in the Chao1 meta-analysis. After excluding this study, we detected a significant between-group difference in Chao1 (*P* = 0.01, *I*^2^ = 0%).

**Figure 4:**
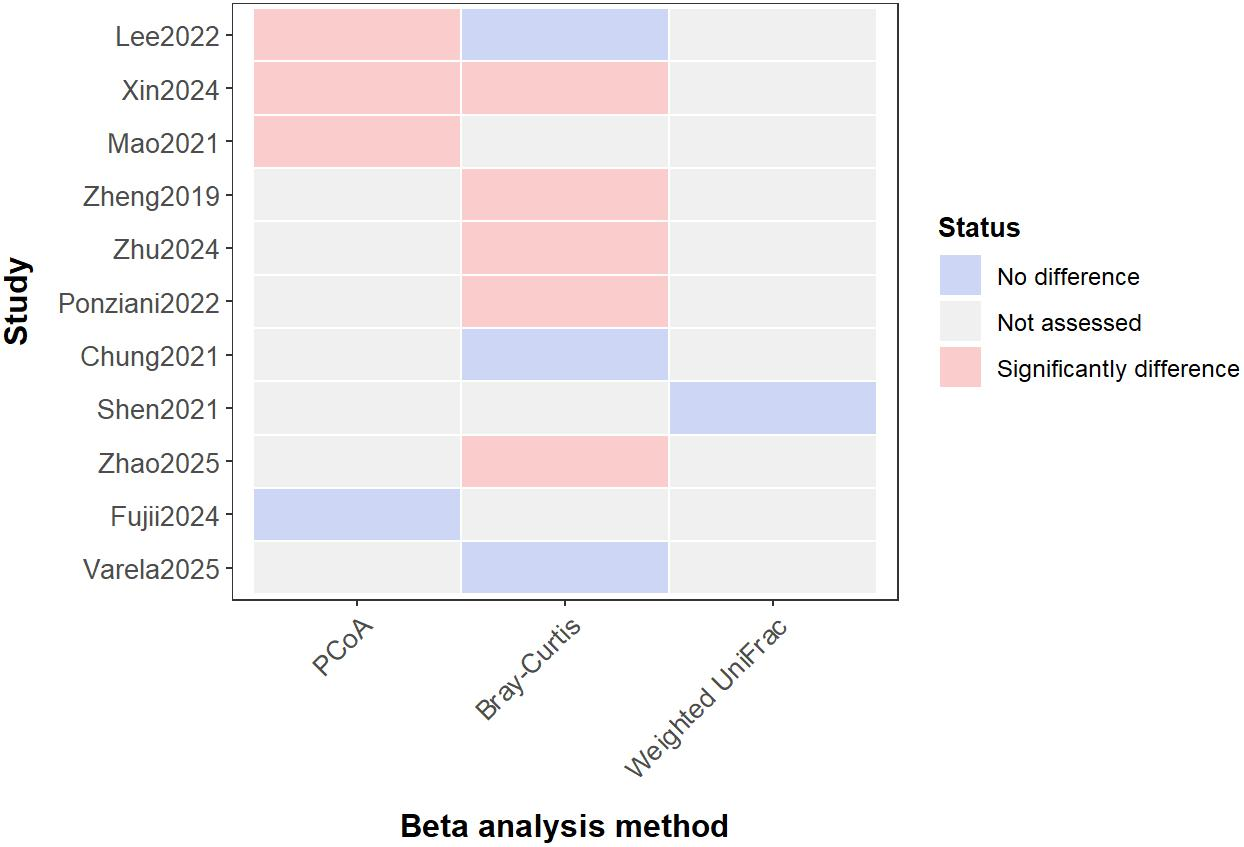
Comparison of beta diversity between patients between R and NR in HCC.

A total of 11 studies provided data on beta diversity. Among these, 2 studies employed two different testing methods. 8 studies used the Bray–Curtis method for the distance calculation, 4 studies utilized Principal coordinate analysis (PCoA) methods, and 1 study employed either weighted or unweighted UniFrac methods (Figure 4). Over 68% (8 of 13) of the studies reported significant differences in *β* diversity, whereas the remaining 5 studies reported no significant differences.

### Gut microbiota profiles of R vs. NR patients at multiple taxonomic levels

**Figure 5:**
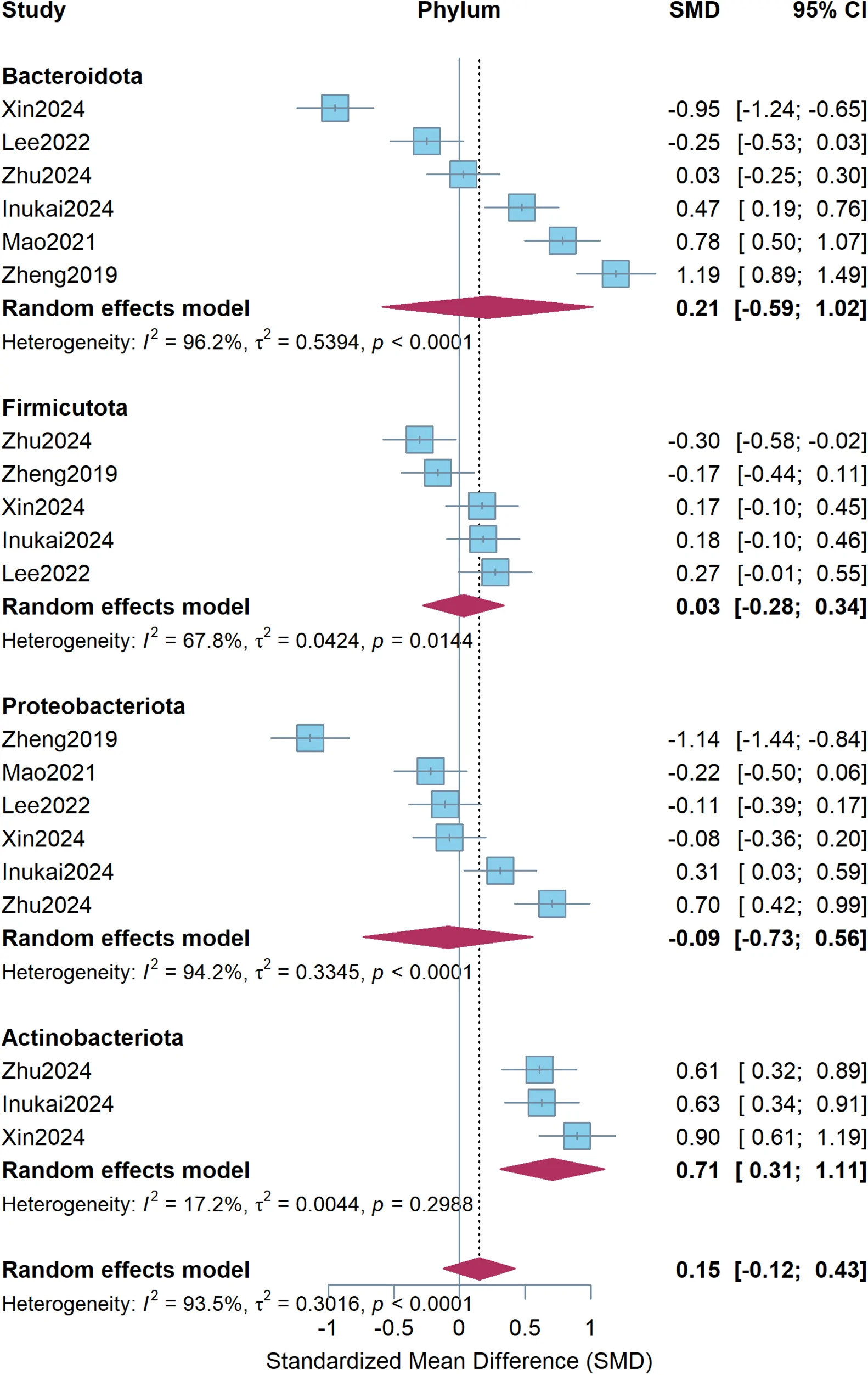
A meta-analysis of the reported microbial taxa at phylum level. Random-effects models were used for meta-analysis. Blue squares and horizontal lines represent single-study SMDs and 95% CIs, while red diamonds reflect subgroup pooled effects. SMD, standardized mean difference; 95% CI, 95% confidence interval; *I*^2^, *I*-square heterogeneity statistic.

**Figure 6:**
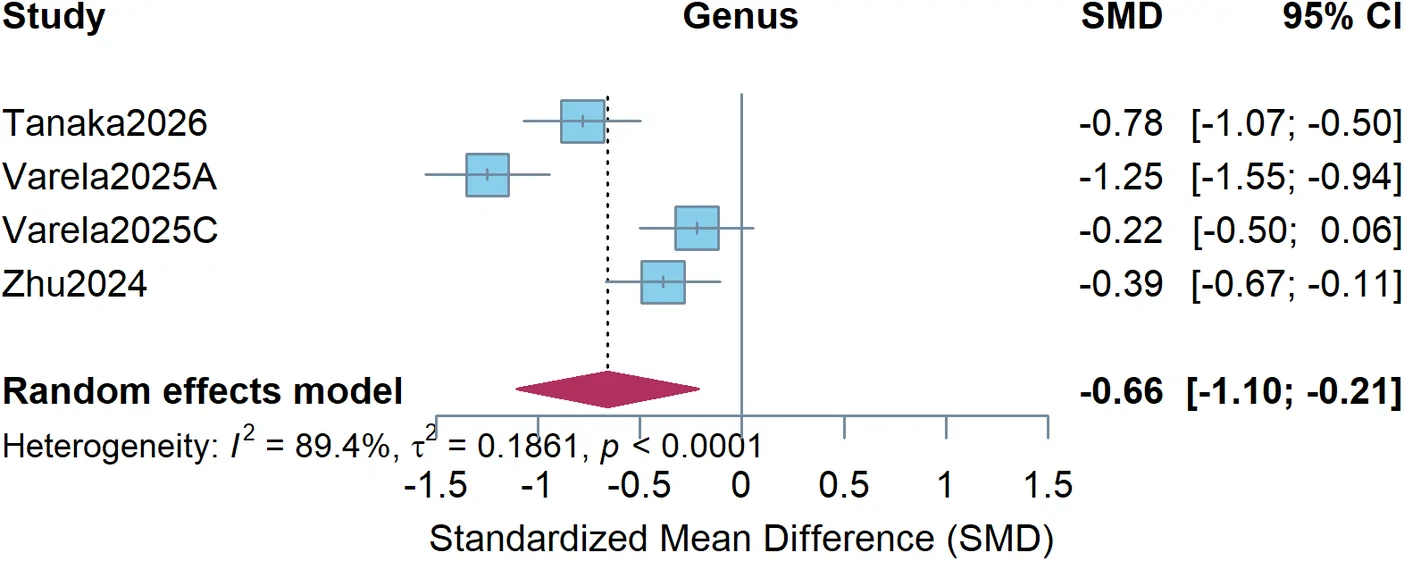
A meta-analysis of *Bacteroides* at genus level. Random-effects models were used for meta-analysis. SMD, standardized mean difference; 95% CI, 95% confidence interval; *I*^2^, *I*-square heterogeneity statistic.

A total of 12 studies examined the relative abundance of gut microbes in patients with R group compared with NR group at the phylum, class, family, genus or species level. We summarize the microbes reported in at least two studies. The changes in 9 phyla, 15 families and 19 genera are summarized. A total of 9 studies utilized the Linear discriminant analysis—effect size (LEfSe) analysis of enriched bacterial taxa between the R group and the NR group. Most of them showed more enriched bacterial taxa in the R group than the NR group (77.7%). We observed some consistent trends: at the genus level, the *Akkermansia* (3/3) were enriched, and the *Bacteroides* (4/4) were depleted; At the phylum level, the *Bacteroidota* (4/6), the *Firmicutota* (3/5) and the *Actinobacteriota* (3/3) were enriched, and the *Proteobacteriota* (4/6) were depleted. We also performed a meta-analysis on over two reported microbial taxa of the studies at the phylum, family, and genus levels. At the phylum level, a significant enrichment of *Actinobacteriota* (SMD = 0.71, 95% CI = 0.31–1.11, *P* < 0.05) was observed. In contrast, the abundance of *Bacteroidota* (SMD = 0.21, 95% CI = −0.59 to 1.02, *I*^2^ = 96.2%, *P* > 0.05), *Firmicutota* (SMD = 0.03, 95% CI = −0.28 to 0.34, *I*^2^ = 67.8%, *P* > 0.05) and *Proteobacteriota* (SMD = −0.09, 95% CI = −0.73 to 0.56, *I*^2^ = 94.2%, *P* > 0.05) were observed no significant difference in analysis (Figure 5). Due to the high heterogeneity observed in taxa belonging to *Bacteroidota, Firmicutota* and *Proteobacteriota*, we stratified all included testing methods into two subgroups and eliminated sources of heterogeneity sequentially. Subgroup analysis revealed that the *I*^2^ value dropped substantially after excluding the studies by Zheng et al. (21)/Zhu et al. (22), but the overall result showed no significant difference (*P* > 0.05; Supplementary Figure 1). At the genus level, only one taxon was reported in more than two studies, allowing comparisons between the R and NR groups. A significant difference was found in the relative abundance of depleted *Bacteroides* (SMD = −0.66, 95% CI = −1.10 to −0.21, *I*^2^ = 89.4%, *P* < 0.05, Figure 6). At the family level, no significant alterations were detected in the abundances of *Bacteroidaceae*, *Lachnospiraceae*, and *Ruminococcaceae* (all *P* > 0.05, Supplementary Figure 2). At the species level, no significant alterations were detected in the abundances of *Faecalibacterium prausnitzii,* and *Escherichia coli* (all *P* > 0.05, Supplementary Figure 3).

### Association of survival benefit with enriched differential bacterial taxa

**Figure 7:**
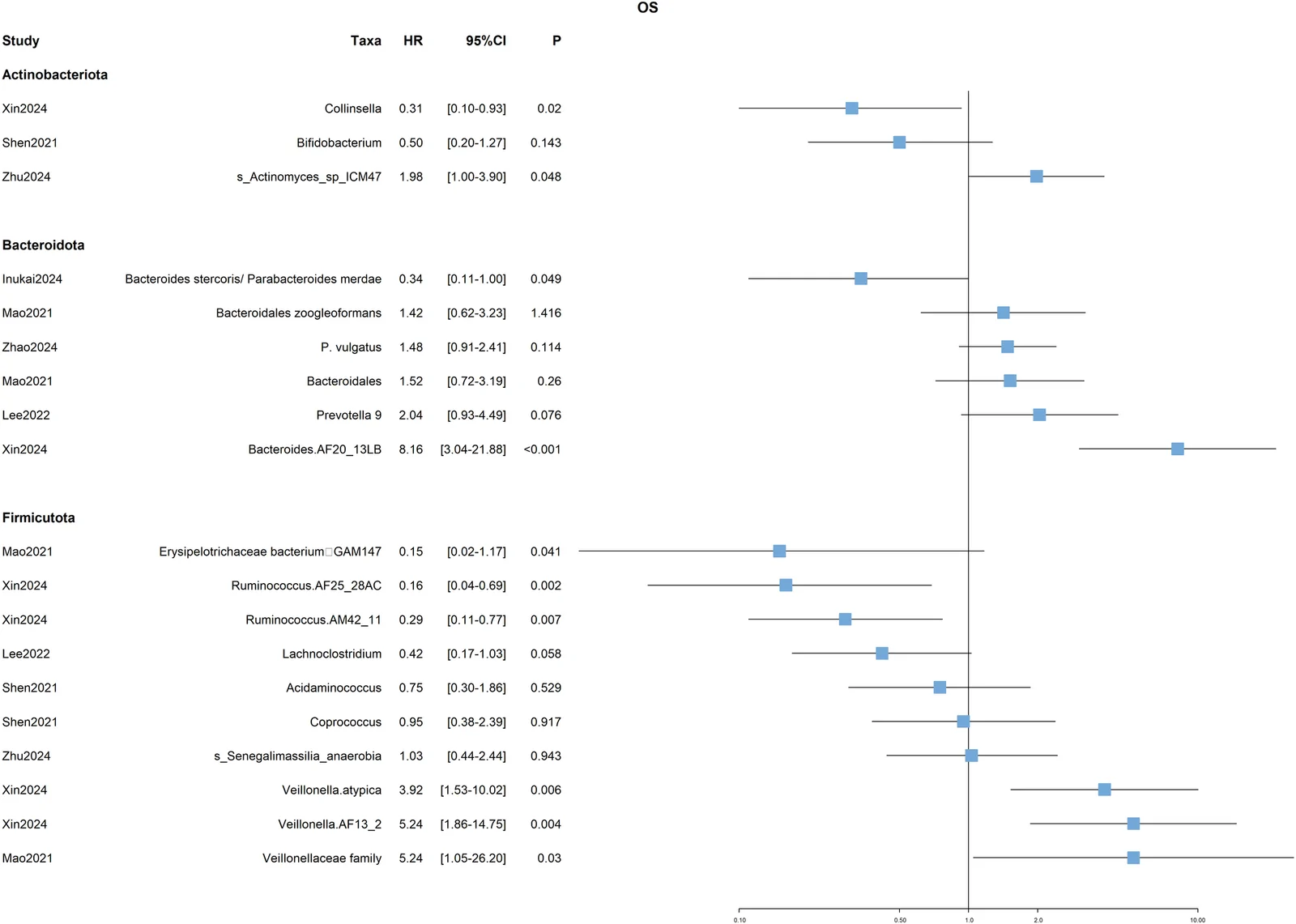
Forest plots of the reported microbial taxa associated with OS. HR, hazard ratio; 95% CI, 95% confidence interval. Taxa are grouped by taxonomic classification (phyla) for visual presentation only. No pooled effect estimate was calculated across biologically distinct microbial taxa. The vertical dashed line represents HR = 1.

**Figure 8:**
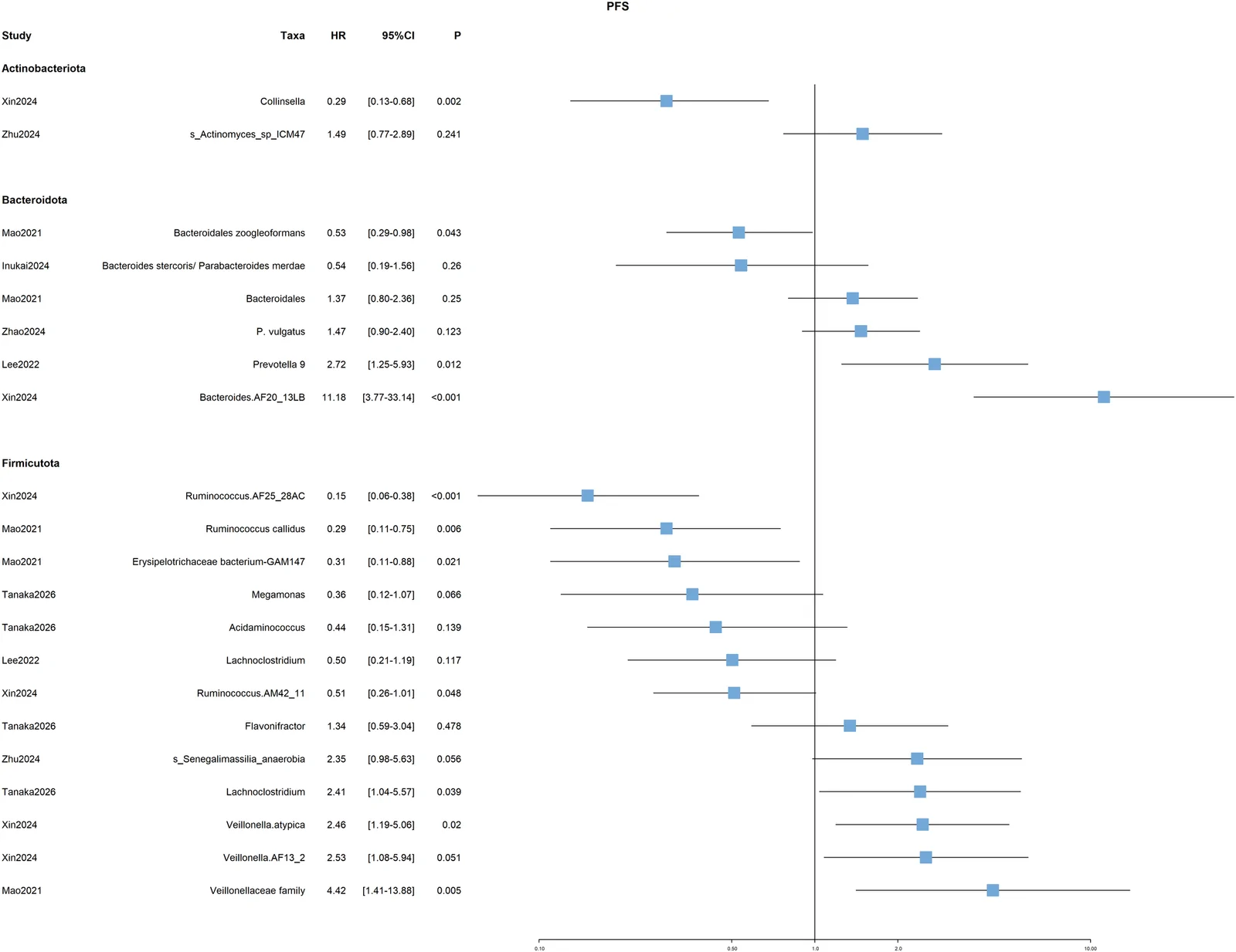
Forest plots of the reported microbial taxa associated with PFS. HR, hazard ratio; 95% CI, 95% confidence interval. Taxa are grouped by taxonomic classification (phyla) for visual presentation only. No pooled effect estimate was calculated across biologically distinct microbial taxa. The vertical dashed line represents HR = 1.

A total of 8 studies provided data on the survival of 335 patients and identified 24 bacterial taxa associated with PFS and OS in the R group and NR group. Three dominant phyla were observed: *Actinobacteriota*, *Firmicutota* and *Bacteroidota* (Supplementary Tables 3, 4). Due to the distinct microbial taxa reported across individual studies, and the fact that most individual taxa were not evaluated in at least two independent cohorts, quantitative meta-analysis of pooled effect sizes for all results was not performed. Instead, study-specific HRs and 95% CIs were summarized descriptively in Figures 7, 8.

We assessed the associations of all bacterial taxa identified in this study with prognostic outcomes using hazard ratio (HR). An HR > 1 corresponds to elevated mortality risk, whereas an HR < 1 corresponds to reduced mortality risk. According to the summary of HR analyses for OS, multiple gut microbial taxa were significantly associated with survival outcomes (*P* < 0.05). Factors associated with reduced mortality risk (HR < 1) included the taxa *Collinsella* (HR = 0.31), *Bacteroides stercoris/Parabacteroides merdae* (HR = 0.34), *Erysipelotrichaceae bacterium GAM147* (HR = 0.15), *Ruminococcus.AF25_28AC* (HR = 0.16), and *Ruminococcus.AM42_11* (HR = 0.29). This suggests their enrichment correlates with reduced mortality risk. Meanwhile, taxa associated with increased mortality risk (HR > 1) included *s_Actinomyces_sp_ICM47* (HR = 1.98), *Bacteroides.AF20_13LB* (HR = 8.16), *Veillonella.atypica* (HR = 3.92), *Veillonella.AF13_2* (HR = 5.24), and *Veillonellaceae* (HR = 5.24), indicating that these microbes are predictive of unfavorable OS outcomes.

Several microbial taxa also exhibited significant correlations with PFS (*P* < 0.05). The protective taxa associated with lower progression risk (HR < 1), namely *Bacteroidales zoogleoformans* (HR = 0.53), *Ruminococcus.AF25_28AC* (HR = 0.15), *Collinsella* (HR = 0.29), *Ruminococcus callidus* (HR = 0.29), and *Erysipelotrichaceae bacterium-GAM147* (HR = 0.31). By contrast, taxa associated with elevated disease progression risk (HR > 1) included *Prevotella 9* (HR = 2.72), *Bacteroides.AF20_13LB* (HR = 11.18), *Lachnoclostridium* (HR = 2.41), and *Veillonella.atypica* (HR = 2.46), suggesting that enrichment of these taxa was significantly correlated with higher risk of disease progression.

### Differences in the metabolism of the fecal microbiota

To clarify the possible influence of gut microbes on metabolism, we qualitatively summarized Kyoto Encyclopedia of Genes and Genomes (KEGG) pathways and metabolite-microbiota correlation results from the included primary studies. Five of these studies conducted correlation analyses between gut microbes, metabolites and KEGG pathways (Supplementary Figure 7 and Supplementary Table 5). Lee et al. (17) reported patients in the R group had higher levels of primary and secondary bile acids, alongside altered metabolites. Fecal ursodeoxycholic acid (UDCA), ursocholic acid (UCA) and murideoxycholic acid (MDCA) were positively correlated with *Lachnoclostridium* and *Ruminococcus gnavus* group (which were enriched in R patients). In contrast, these secondary bile acids were negatively correlated with *Prevotella 9* (the dominant genus in NR patients).

Mao et al. (18) further annotated metagenomic sequencing data using the KEGG database, and identified 29 orthologs (KOs) enriched in the R group (primarily energy metabolism pathways) as well as 4 KOs enriched in the NR group (primarily amino acid metabolism pathways). MetaCyc analysis showed that R was dominated by bacterial reproduction-related pathways.

Zheng et al. (21) identified 189 KOs enriched in the responder group, with 18 R-enriched species. Zhu et al. (22) analyzed multi-omics data and found three core shared pathways (KO00250, KO00910, KO00750) across microbes and metabolites, with KO00250 being the most critical. KO00250 and KO00910 were present in the NR group. Zhao et al. (25) found that in the NR group, the abundances of *P. vulgatus*-related genes iorA (K00179) and iorB (K00180) were elevated, whereas ipdC (K04103) was decreased. Meanwhile, plasma indole-3-acetic acid (IAA) concentrations were negatively correlated with fecal abundance of *P. vulgatus*.

### Gut microbiota and metabolism for the differential diagnosis and prediction of prognosis

A total of five included studies collected clinical data of HCC patients stratified by treatment response, and further investigated the correlations between these clinical indicators and gut microbiota (Supplementary Table 6). Lee et al. (17) revealed that a microbial signature (enriched *Lachnoclostridium* and depleted *Prevotella 9*) independently predicted favorable overall survival (OS). Meanwhile, gut microbiota abundance and fecal UDCA were linked to survival. Mao et al. (18) investigated the sixteen differential taxa were found between mild and severe diarrhea groups; taxa within *Firmicutota* phylum differed across groups, while *Prevotellamassilia timonensis* was enriched in patients with severe diarrhea. Further analysis showed that taxa enriched in the NR group were linked to elevated bile acid, bilirubin and impaired liver function. *Eubacterium hallii* was more abundant in patients with large tumors (≥5 cm).

By contrast, *Faecalibacterium prausnitzii*, which was enriched in the R group, was negatively correlated with tumor number and stage. Consistent with the findings reported by Tanaka et al. (23), high levels of *Faecalibacterium* (*P* = 0.016) were associated with favorable prognosis for patients. Zhu et al. (22) found that *Clostridium scindens* and *Clostridia* bacterium UC5_1_1D1 were strongly correlated with total bilirubin and bile acids. Multiple tumor-related factors, tumor size, and vascular invasion, significantly influenced the distribution of *s_Candidatus Avimonas narfia*. Shen et al. (24) reported that high baseline levels of *Acidaminococcus* and *Coprococcus* were linked to no antibiotic use in the 4 weeks prior to immunotherapy and absence of prior systemic treatment.

### Sensitivity analysis and analysis of publication bias

Leave-one-out sensitivity analyses were conducted across all pooled meta-analyses (alpha-diversity and taxonomic-level comparisons) to assess the impact of individual studies on effect sizes, *P*-values and heterogeneity. Non-significant between-group differences remained consistent for most alpha diversity metrics after removing individual studies one at a time. However, after excluding the Inukai 2024 study (NOS = 7), the pooled analysis of the Chao1 index yielded a statistically significant difference (SMD = 0.43, 95% CI 0.09–0.77, *P* = 0.014), and inter-study heterogeneity was fully eliminated. Removing other trials did not substantially alter the overall pooled results. Given that the detection method adopted in the Inukai 2024 study differed from those of other included studies, the findings regarding the Chao1 index should be interpreted with caution.

For taxonomic levels analyses, leave-one-out sensitivity showed modest changes for most pooled estimates, but for phyla including *Firmicutota* and *Proteobacteriota*, individual studies exclusion produced large *I*^2^ reductions, while the direction and statistical significance of pooled effects remained unchanged. These findings indicate that heterogeneity was largely driven by single studies, and these results should be interpreted cautiously (see Results section). Funnel plots were generated for the pooled SMD analysis. Egger's regression test yielded an intercept of 15.801 (*P* = 0.6655), indicating no statistically significant evidence of funnel-plot asymmetry. Nevertheless, visual asymmetry was observed in the funnel plot, and nearly all included studies had high standard errors, representing small-sample investigations. Trim-and-fill analysis suggested one hypothetically missing study; after imputation, the pooled SMD changed only slightly, implying that our main findings remained relatively robust (Supplementary Figures 8, 9).

## Discussion

HCC is far more prevalent in Asia than in Western countries and represents the third most common cause of cancer death in the Asia-Pacific area. Although emerging therapies and ongoing research have yielded modest prognostic gains, patients still face poor survival, with a median survival below 2 years. As immune checkpoint inhibitors are monoclonal antibodies that target inhibitory immune axes such as programmed cell death protein 1/programmed death-ligand 1 (PD-1/PD-L1) and cytotoxic T-lymphocyte antigen-4 (CTLA-4) to reverse immunosuppression, they have become a critical therapeutic strategy for advanced HCC (28). However, patient clinical outcomes show substantial heterogeneity, underscoring an urgent demand for reliable biomarkers to precisely predict responses to immunotherapy. Distinct subsets of the gut microbiota exert divergent effects: some exacerbate inflammation and DNA lesions linked to tumorigenesis, while others facilitate robust anti-tumor immune activation and thereby amplify the clinical benefits of cancer therapeutics, immunotherapy in particular (29). Preclinical evidence indicates that faecal microbiota transplantation (FMT) and *Akkermansia* supplementation may rescue impaired therapeutic responses to ICIs (30). Accordingly, modulation of the gut microbiome may serve both as a predictive biomarker and a promising therapeutic strategy for the clinical management of HCC.

We noticed a recently published review summarizing gut microbiota biomarkers in hepatocellular carcinoma patients treated with immune checkpoint inhibitors (31). Compared with this prior review, our analysis included nearly twice the number of eligible studies and utilized more available data to reach more robust and comprehensive conclusions.

Therefore, this study seeks to investigate differences in gut microbiome among HCC patients with divergent therapeutic responses, and further correlate these microbial profiles with corresponding prognostic outcomes.

Eventually, 13 studies were included in this analysis. First, we analyzed the alpha diversity of gut microbiota based on the included 8 studies. Only one study reported a significant difference in the Shannon index between groups. Consistently, meta-analysis of Shannon, Simpson, and ACE index revealed no obvious disparities in overall gut microbial diversity between responders (R) and non-responders (NR). In contrast, significant intergroup differences were observed for the Chao 1 index after excluding studies with high heterogeneity. The elevated heterogeneity was attributed to inconsistent detection methods used in this single trial compared with the other three investigations. Therefore, the findings regarding the Chao1 index should be interpreted with caution.

Nevertheless, significant differences in beta diversity were detected in more than 68% of the included studies. This discrepancy may be explained by the distinct biological implications of alpha and beta diversity. Alpha diversity quantifies the total richness and uniformity of all microbial species within a single sample, which is largely shaped by long-term fixed factors including regional dietary habits, underlying cirrhosis status and chronic viral hepatitis background (32, 33). All enrolled HCC patients had consistent baseline profiles, resulting in non-significant alterations in gut microbial alpha diversity. Accordingly, microbial richness and evenness were comparable between ICI responders and non-responders. Furthermore, variations in alpha diversity for most bacterial taxa disappeared after 6–8 weeks; this time point may represent the threshold at which disparities in long-term immunotherapy efficacy emerge in patients with HCC (18, 24). In contrast, *β*-diversity characterizes the overall compositional structure of gut microbiota (34). Even when the total quantity of intestinal microbes remains similar between cohorts, dramatic shifts in the relative abundance of immune-modulatory core taxa can fundamentally remodel hepatic anti-tumor immunity (8), leading to significant separation in beta diversity rather than alpha diversity. Future research ought to focus not only on baseline gut bacterial and fungal diversity but also on alterations in gut microbiota diversity 6–8 weeks after immunotherapy.

We further systematically summarized differential gut microbial taxa reported across all eligible studies, covering alterations across 9 phyla, 19 genera and 15 families. The majority of these discriminatory microbes were more abundant in ICI responders (R group) relative to non-responders (NR group). Taxon-specific patterns exhibited clear stratification: at the genus level, *Akkermansia* (3/3) were significantly enriched, *Bacteroides* was consistently depleted (4 out of 4 studies). At the phylum level, *Bacteroidota* (4/6), *Firmicutota* (3/5), and *Actinobacteriota* (3/3) were significantly enriched in responders, whereas *Proteobacteriota* (4/6) presented lower abundance. Owing to the high heterogeneity of *Firmicutota* and *Proteobacteriota*, the pooled meta-analysis only reached statistical significance after excluding the studies conducted by Zhu and Zheng. Patients receiving Lenvatinib were included in Zhu's cohort, whereas the primary etiology of HCC was unclear for participants in Zheng's study. Meanwhile, unlike other studies, both of these two studies adopted whole-genome shotgun sequencing for microbial profiling. These three factors might introduce inter-study heterogeneity, suggesting that the conclusions from this research require prudent interpretation. Consistent with Huang et al. (35), *Patesibacteriota*, *Proteobacteriota*, *Bacteroidota*, *Firmicutota* and *Actinobacteriota* together accounted for 92.88% of all detected bacterial taxa. Among them, *Bacteroidota*, *Firmicutota* and *Actinobacteriota* were significantly enriched in patients with HCC compared with healthy individuals. Consistent with Wu et al. (36), who found that *Akkermansia* abundance declines as HCC progresses, we observed significant enrichment of *Akkermansia* in immunotherapy responders. Functionally, *Akkermansia* reinforces intestinal barrier integrity, lowers serum lipopolysaccharide (LPS) and bile acid metabolites, and suppresses m-MDSCs and M2 macrophage accumulation. This bacterium alleviates hepatic steatosis and restrains tumor proliferation, with enhanced anti-tumor efficacy when combined with anti-PD-1 therapy. Meanwhile, animal experiments have demonstrated that FMT from non-responders combined with oral supplementation of beneficial taxa such as *Akkermansia* increases the infiltration of CCR9^+^, CXCR3^+^ and CD4^+^ T lymphocytes within the mouse tumor microenvironment. This restores the efficacy of PD-1 blockade in an interleukin-12-dependent manner, accompanied by enhanced CD8^+^ T-cell infiltration and tumor-cell apoptosis (30). No consistent statistically significant shifts in microbial abundance were observed at the family taxonomic rank. This is related to the insufficient number of studies we included.

HR analyses revealed that distinct taxa belonging to the same phylum exerted opposing prognostic effects on OS and PFS in HCC patients, especially taxa from the phyla *Bacteroidota* and *Firmicutota*. Clinical outcomes of HCC patients were more strongly associated with *Firmicutota*; enrichment of this phylum correlated with better immunotherapy responses and improved survival outcomes (18). Of note, this intra-phylum heterogeneity indicates that higher-level taxonomic membership should not be interpreted as evidence of a common prognostic effect. Small sample sizes may reduce statistical power and increase the risk of false-positive associations. Therefore, we reviewed extensive literature to support our results. Chung et al. (16) reported that the *Firmicutota*/*Bacteroidota* ratio among all responders varied between 0.54 and 1.44, with a mean value of 0.88. By contrast, 66.7% of non-responders displayed markedly skewed ratios (either <0.5 or >1.5). Zhang et al. (37) confirmed that a high *Firmicutota*/*Bacteroidota* ratio and enriched *Proteobacteriota* collectively promote the advancement of HBV-induced liver disorders. These findings strongly support the hypothesis that an extreme imbalance in the *Firmicutota*/*Bacteroidota* ratio could serve as a biomarker for poor nivolumab treatment response.

Meanwhile, one of the included studies further verified that colonization with a cocktail of six *Bacteroidota* strains in PFS mice accelerated tumor progression relative to control animals. Significant differences in survival were observed at day 90 following hydrodynamic tail-vein injection (*P* < 0.05) (27). Consistently, following FMT, responders exhibited dominant *Firmicutota* and marked depletion of *Bacteroidota* (38). Furthermore, prior melanoma research demonstrated that elevated *Bacteroides* was negatively associated with prolonged PFS in patients receiving CTLA-4 blockade (39). The role of *Bacteroidota* in tumorigenesis and immunotherapy remains controversial, which may stem from differences in cancer types and heterogeneous species composition within this phylum. Future targeted research is therefore warranted to clarify the divergent prognostic effects of distinct *Bacteroidota* members.

Notably, functional divergence was also prominent within the phylum *Firmicutota*. For instance, *Ruminococcus spp.* (including *Ruminococcus AM42_11*, *Ruminococcus AF25_28AC* and *Ruminococcus callidus*) and *Veillonella* lineages (*Veillonella atypica*, *Veillonella* AF13_2, *Veillonellaceae*) show divergent outcomes: *Ruminococcus* correlates with favorable prognosis, whereas *Veillonella* is associated with disease progression. Other studies further validated that *Ruminococcus* abundance decreased significantly in advanced HCC patients, whereas *Veillonella* was correlated with early HCC recurrence (40, 41). One study investigating gut microbiota profiles in HCC patients with or without macrovascular invasion (MVI) revealed enrichment of *Ruminococcus* in patients without MVI, whereas abundant *Veillonella*, *Prevotella_9*, and *Bacteroides* were detected in those with MVI (42). Such intra-taxon functional heterogeneity underscores that species-level microbial profiling, instead of crude phylum-level analysis, is required for precise prognostic stratification. Collectively, these results hint that microbiota-modulating interventions at precancerous stages might hold theoretical promise for delaying HCC progression, though this hypothesis remains to be verified in clinical studies.

With respect to the gut microbiota metabolic differences between responder and non-responder patients with HCC, three studies mentioned *Lachnoclostridium, Ruminococcus gnavus* and *Megamonas*, but there were some differences in the research results. Lee et al. (17) indicated that fecal UDCA, UCA and MDCA were positively correlated with *Lachnoclostridium* and *Ruminococcus gnavus* enriched in R group. Chung et al. (16) reported a relatively high relative abundance of *Megamonas* in the non-responder group. Instead, Tanaka et al. (23) revealed patients with higher abundance of *Acidaminococcus* and *Megamonas* showed significantly prolonged PFS. Moreover, a higher prevalence of *Ruminococcus_gnavus* and *Lachnoclostridium* was observed in the NR group, and the abundance of the microbiota was negatively correlated with PFS. Such inconsistency may be attributed to the dual regulatory roles of bile acids in tumor immunity (43), heterogeneous strain functions within the same genus, and divergent clinical baseline characteristics of HCC patients enrolled in different cohorts. Meanwhile, another recent study reported that *Megamonas* was enriched in MASLD patients with high BMI. In patients with elevated CAC scores (≥100 AU), the abundances of *Ruminococcus gnavus*, *Bacteroides* and *Lachnoclostridium* were increased, accompanied by reduced levels of several short-chain fatty acid (SCFA)-producing bacteria (44). Notably, a large cohort of over 1,000 Chinese participants recently defined an obesity-associated gut enterotype dominated by Megamonas, which accumulates in obese people. Mouse experiments and functional assays further revealed that these microbes drive obesity via suppressed fatty acid transport, myoinositol breakdown and enhanced lipid uptake (45). Dysregulated lipid metabolism facilitates the late-stage formation of tumor microenvironments, establishes an immunosuppressive niche, and directly fuels HCC progression (46). To reduce such inconsistencies in future research, standardized enrollment criteria with rigorous adjustment for confounders including BMI, metabolic status and bile acid profiles are required. Multi-omics integration combined with germ-free animal validation is recommended to clarify causal relationships between key microbes, lipid/bile acid metabolism and therapeutic outcomes. Additionally, large independent multi-center cohorts and multivariate statistical models are necessary to validate microbial biomarkers and eliminate inter-cohort heterogeneity.

Our results revealed that taxa enriched in responders exhibit robust bile acid metabolic capacities. For example, *Erysipelotrichaceae* bacterium-GAM147 acts as a butyrate-producing commensal bacterium, significantly associated with prolonged survival in patients receiving immunotherapy. *Lachnospiraceae* and *Erysipelotrichaceae* are beneficial commensals that ferment dietary fiber to generate SCFAs, especially butyrate. Beyond SCFA biosynthesis, these taxa also mediate bile acid turnover, a metabolic axis capable of shaping host responsiveness to ICI treatment. Mechanistically, butyrate itself exerts anti-tumor activity by restraining HCC proliferation and metastasis through calcium signaling cascade activation and the upregulation of intracellular reactive oxygen species (ROS) (47).

Although the family *Ruminococcaceae* exhibited a trend of enrichment in treatment responders without statistical significance, Mendelian randomization analyses have validated its protective role against HCC development (48). Functionally, *Ruminococcaceae* is essential for secondary bile acid synthesis; its depletion may aggravate intestinal inflammation and break down host immune homeostasis. Consistent with this, subsequent FMT experiments demonstrated that *Ruminococcaceae* abundance was markedly elevated after transplantation, which reinforced anti-tumor immunity in both mouse models and immunotherapy-treated patients by boosting tumor-infiltrating IFN-*γ*^+^, CD8^+^ T cells (40, 49). A high abundance of *Akkermansia muciniphila* was also observed in R, with robust anti-inflammatory properties. Meanwhile, the commensal *A. muciniphila* and *Ruminococcaceae* support host health by inhibiting the rise of intestinal permeability and mitigating systemic immunosuppression. Furthermore, metabolomic analyses reveal that *Akkermansia* reshapes host bile acid metabolism and elevates serum Tauroursodeoxycholic acid. Given Tauroursodeoxycholic acid's immunosuppressive effects on HCC, this genus may potentiate anti-HCC PD-1 blockade by modulating bile acid profiles (50).

In NR group, both *Actinomyces* and *Veillonella* were potential prognostic markers that were negatively associated with immunotherapy. Both of them linked to butyric acid (and derivatives) and cyclohexanecarboxylic acid ethyl ester, as well as to higher levels of neutrophils (51). Additionally, non-responder melanoma patients have been found to be enriched in *Veillonella atypica* (52). Other studies have reported that elevated butyrate concentrations correlate with the relative abundance of certain taxa prevalent in non-responders, including *Actinobacteriota, Streptococcus* and *Veillonella* (53). Recent research further demonstrates that butyrate (along with propionate) impairs the efficacy of immunotherapy. The underlying mechanism may stem from the immunomodulatory properties of butyrate. Specifically, butyrate promotes the differentiation of regulatory T cells (Tregs), suppresses the accumulation of memory T cells, and downregulates the expression of inducible co-stimulatory molecules on T cells; it also blunts the anti-tumor activity of ipilimumab by hindering the recruitment of response-associated CD4^+^ T cells (54). Therefore, identifying taxa associated with prognosis is of great significance. In the future, oral supplementation or FMT targeting these beneficial microbes might theoretically help reinforce anti-tumor immunity and mitigate therapeutic resistance, potentially improving anti-tumor efficacy. Nevertheless, abundant preclinical and clinical trials are still required to further validate such therapeutic strategies.

In summary, gut microbiota exert no unidirectional control over tumor immune escape via microbial metabolites (55). They exhibit dual functions, with their homeostatic balance dictating tumor immune escape outcomes. Beneficial taxa enriched in immunotherapy responders (*Akkermansia*, *Actinobacteriota, Firmicutota*) shape anti-tumor immunity via short-chain fatty acids, secondary bile acids and tryptophan metabolites (56). Butyrate enhances dendritic cell antigen presentation, promotes CD8^+^ cytotoxic T cell activation and memory T cell formation, and relieves local immunosuppression in hepatic tumor microenvironments. Conversely, *Proteobacteriota* (depleted in responders) may impair intestinal barrier function, induce low-grade hepatic inflammation and recruit immunosuppressive cells to establish an ICI-resistant “cold-tumor” phenotype (57). Large scale multicenter studies are needed to validate additional associations between microbiota and ICI efficacy in HCC.

This study also preliminarily explored the correlations between the gut microbiome, immune checkpoint inhibitor-related adverse events, and routine clinical laboratory parameters. First, the two groups exhibited significant differences in the abundance of gut microbiomes associated with irAEs. *Prevotellamassilia timonensis* (*Bacteroidota* phylum) was enriched in patients with severe diarrhea and may serve as a biomarker for severe immunotherapy-related colitis. While the *Firmicutota* phylum was significantly enriched in the mild diarrhea group, we inferred that taxa from the *Firmicutota* phylum may serve as protective factors against immunotherapy-induced toxicity. Besides, *Akkermansia* and low fecal calprotectin concentration were associated with disease control. Notably several studies have suggested a positive correlation between the occurrence of irAEs and patient survival (58, 59). Nevertheless, severe diarrhea impairs patients’ nutritional status and treatment adherence. Whether gut microbiota can serve as a therapeutic target to alleviate diarrhea and simultaneously prolong patient survival warrants further investigation.

Second, the two groups exhibited differences in gut microbiomes associated with tumor burden. *Eubacterium hallii* displayed higher abundance in patients with large tumors (≥5 cm), whereas *Faecalibacterium prausnitzii*—a taxon enriched in immunotherapy responders—exhibited negative correlations with tumor count and tumor stage, implying its protective potential against progressive tumor burden. Consistent with our findings, previous studies have demonstrated that *Eubacterium* and *Faecalibacterium* are linked to butyrate and lipopolysaccharide biosynthesis. These two genera maintain intestinal integrity and exert anti-inflammatory and immunomodulatory effects through SCFAs, making them promising biomarkers for distinguishing HCC patients with persistent HCV viremia (60). Some studies have reported that co-culturing *Akkermansia muciniphila* with butyrate-producing bacteria including *Faecalibacterium prausnitzii* and *Eubacterium hallii* markedly elevates butyrate production. This indicates that targeted microbial co-culture strategies can boost the proliferation of other anti-inflammatory gut commensals (61, 62).

Third, the two groups exhibited differences in gut microbiomes associated with liver function markers. *Clostridium scindens* and *Clostridia* bacterium UC5_1_1D1 showed strong positive correlations with total bilirubin and bile acids, further revealing close crosstalk between the dysregulated microbiota of non-responders and aberrant bile acid–hepatic metabolic profiles. Animal experiments have demonstrated the elevated relative abundance of *Clostridium* in Nucleotide-binding oligomerization domain-containing 2 (Nod2)-deficient mice. Loss of Nod2 function further upregulates hepatic genes involved in inflammation, lipogenesis, cholesterol metabolism and liver fibrosis (63, 64). In addition, major liver function markers and tumor pathological features (aspartate aminotransferase, alanine aminotransferase, gamma-glutamyl transferase, tumor size and vascular invasion) exert profound influences on gut microbial profiles, which is evidenced by the altered distribution of *Candidatus Avimonas narfia*. Collectively, these data demonstrate that gut microbiota signatures are not only tied to immunotherapy response but also dynamically associated with tumor progression characteristics and liver injury indices, forming a complex regulatory network linking intestinal flora, bile acid metabolism, hepatic function and tumor progression. The clinical response and survival advantages of ICI treatment rely on gut microbial diversity as well as taxonomical communities with differential enrichment in the intestinal tract.

### Limitations

To elucidate the role of gut microbiota in the pathogenesis of HCC among patients with and without treatment response, we conducted a systematic literature search and synthesized ample published datasets; however, most original studies failed to provide detailed raw data correlating bacterial abundance with patient survival. The KEGG-related findings were extracted from original publications rather than generated in our study. Meanwhile, the present meta-analysis has several inherent limitations. First, geographic location, ethnicity, dietary habits, smoking status, medication-related factors (antibiotic use, proton-pump inhibitor use, probiotic supplementation), HCC etiology and baseline disease severity are all confounding factors capable of reshaping gut microbial composition (Supplementary Table S7). Second, hepatocellular carcinoma represents a highly heterogeneous malignancy whose progression is governed by a complex array of variables, which may partially account for the inconsistent microbial signatures observed across independent cohorts. Large-scale, stratified studies with expanded sample sizes are therefore required to validate these preliminary findings. Third, the gut microbial ecosystem encompasses not only bacteria but also viruses and archaea; further research is warranted to clarify whether these non-bacterial microbes exert critical synergistic effects in modulating immunotherapeutic responses in HCC patients.

Methodological differences in fecal sample processing, sequencing platforms (16S rRNA and shotgun metagenomics), and radiological criteria (RECIST/mRECIST) may explain inconsistent microbial findings across cohorts. Further studies with larger sample sizes are needed to obtain more reliable results. Additionally, as we performed multiple independent meta-analyses on a large set of microbial taxa without applying global multiple-testing correction, type-I error cannot be fully ruled out. Meanwhile, given the observational design and limited number of included studies, causal inference linking specific microbial taxa to ICI treatment response is limited. In addition, reported KEGG pathways may not faithfully mirror *in-vivo* microbial metabolic activity, as they are not direct measurements of gene expression or metabolites. Prospective multicenter studies are required to validate the clinical applicability of these candidate microbial biomarkers.

Despite these limitations, findings from this meta-analysis point toward gut microbiota as a potentially actionable intervention target. The taxa and metabolic pathways we identified may offer clues for developing modulatory strategies to improve treatment outcomes among patients with hepatocellular carcinoma.

## Conclusion

HCC patients with varied treatment responses exhibit characteristic shifts in gut flora. Compositional and functional variations of intestinal microbes serve as potential indicators of disease status, prognostic predictors, and references for clinical therapeutic decisions. Therefore, gut microbiota may represent a promising biomarker and potential therapeutic target for future HCC immunotherapy.

## Data Availability

The original contributions presented in the study are included in the article/Supplementary Material, further inquiries can be directed to the corresponding authors.

## References

[1] Mauro E De Castro T Zeitlhoefler M Sung MW Villanueva A Mazzaferro V. Hepatocellular carcinoma: epidemiology, diagnosis and treatment. JHEP Rep. (2025) 7(12):101571. 10.1016/j.jhepr.2025.101571 41244300 PMC12615749

[2] Hwang SY Danpanichkul P Agopian V Mehta N Parikh ND Abou-Alfa GK. Hepatocellular carcinoma: updates on epidemiology, surveillance, diagnosis and treatment. Clin Mol Hepatol. (2025) 31(Suppl):S228–54. 10.3350/cmh.2024.0824 39722614 PMC11925437

[3] Meyers BM Lim HJ Brahmania M Liu DM Tam VC McLeod D. Role of immune checkpoint inhibitor combinations in resectable and unresectable, embolization-eligible hepatocellular carcinoma. Ther Adv Med Oncol. (2025) 17:17588359251357719. 10.1177/17588359251357719 40727882 PMC12301614

[4] Singal AG Salem R Pinato DJ Pillai A. Advances in locoregional and systemic treatments for hepatocellular carcinoma. Gastroenterology. (2025) 169(4):585–99. 10.1053/j.gastro.2025.03.047 40320088 PMC13421180

[5] Dhyani A Harding JJ. Immune checkpoint blockade and transarterial chemoembolization in liver-limited hepatocellular carcinoma: new questions at the Dawn of a new era. J Immunother Cancer. (2025) 13(8):e012658. 10.1136/jitc-2025-012658 40866293 PMC12410684

[6] Chen Z Liao S Wu S Chen S Tang Q Zhou L. The gut‒liver axis in liver disease: molecular mechanisms and therapeutic targets. MedComm. (2025) 6(11):e70458. 10.1002/mco2.70458 41235399 PMC12606024

[7] Wang J Wang X Zhuo E Chen B Chan S. Gut-liver axis in liver disease: from basic science to clinical treatment (review). Mol Med Rep. (2024) 31(1):10. 10.3892/mmr.2024.13375 39450549 PMC11541166

[8] Huang M Ji Q Huang H Wang X Wang L. Gut microbiota in hepatocellular carcinoma immunotherapy: immune microenvironment remodeling and gut microbiota modification. Gut Microbes. (2025) 17(1):2486519. 10.1080/19490976.2025.2486519 40166981 PMC11970798

[9] Yoon SJ Han SK Kim TS Suk KT Choi DH Kim YD. The crosstalk between gut microbiota and microbiota-derived metabolites in hepatocellular carcinoma. Crit Rev Microbiol. (2025) 51(6):1315–29. 10.1080/1040841X.2025.2501590 40468702

[10] Yan Z-B Han C-L Jia J-S Li H Lu D-H Cao Q-H. The landscape of gut microbiota in hepatocarcinogenesis: a comprehensive review of pathogenesis and therapeutic interventions. Int J Surg. (2026) 112(1):1673–95. 10.1097/JS9.0000000000003511 40990659 PMC12825765

[11] Pamungkas KMN Lesmana Dewi PIS Alamsyah AZ Dewi NLPY Dewi NNGK Mariadi IK. Microbiome dysbiosis and immune checkpoint inhibitors: dual targets in hepatocellular carcinoma management. World J Hepatol. (2025) 17(7):106810. 10.4254/wjh.v17.i7.106810 40747222 PMC12308605

[12] Moher D Liberati A Tetzlaff J Altman DG. Preferred reporting items for systematic reviews and meta-analyses: the PRISMA statement. PLoS Med. (2009) 6(7):e1000097. 10.1371/journal.pmed.1000097 19621072 PMC2707599

[13] Shi J Luo D Wan X Liu Y Liu J Bian Z. Detecting the skewness of data from the five-number summary and its application in meta-analysis. Stat Methods Med Res. (2023) 32(7):1338–60. 10.1177/09622802231172043 37161735

[14] Stang A. Critical evaluation of the Newcastle-Ottawa scale for the assessment of the quality of nonrandomized studies in meta-analyses. Eur J Epidemiol. (2010) 25(9):603–5. 10.1007/s10654-010-9491-z 20652370

[15] Inukai Y Yamamoto K Honda T Yokoyama S Ito T Imai N. Intestinal microbiome associated with efficacy of atezolizumab and bevacizumab therapy for hepatocellular carcinoma. Cancers. (2024) 16(9):1675. 10.3390/cancers16091675 38730627 PMC11083184

[16] Chung M-W Kim M-J Won EJ Lee YJ Yun Y-W Cho SB. Gut microbiome composition can predict the response to nivolumab in advanced hepatocellular carcinoma patients. World J Gastroenterol. (2021) 27(42):7340. 10.3748/wjg.v27.i42.7340 34876793 PMC8611200

[17] Lee P-C Wu C-J Hung Y-W Lee CJ Chi C-T Lee I-C. Gut microbiota and metabolites associate with outcomes of immune checkpoint inhibitor–treated unresectable hepatocellular carcinoma. J Immunother Cancer. (2022) 10(6):e004779. 10.1136/jitc-2022-004779 35738801 PMC9226985

[18] Mao J Wang D Long J Yang X Lin J Song Y. Gut microbiome is associated with the clinical response to anti-PD-1 based immunotherapy in hepatobiliary cancers. J Immunother Cancer. (2021) 9(12):e003334. 10.1136/jitc-2021-003334 34873013 PMC8650503

[19] Ponziani FR De Luca A Picca A Marzetti E Petito V Del Chierico F. Gut dysbiosis and fecal calprotectin predict response to immune checkpoint inhibitors in patients with hepatocellular carcinoma. Hepatol Commun. (2022) 6(6):1492–501. 10.1002/hep4.1905 35261212 PMC9134810

[20] Xin Y Peng G Song W Zhou X Huang X Cao X. Gut microbiota as a prognostic biomarker for unresectable hepatocellular carcinoma treated with anti-PD-1 therapy. Front Genet. (2024) 15:1366131. 10.3389/fgene.2024.1366131 39421302 PMC11484251

[21] Zheng Y Wang T Tu X Huang Y Zhang H Tan D. Gut microbiome affects the response to anti-PD-1 immunotherapy in patients with hepatocellular carcinoma. J Immunother Cancer. (2019) 7:193. 10.1186/s40425-019-0650-9 31337439 PMC6651993

[22] Zhu C Zhang C Wang S Xun Z Zhang D Lan Z. Characterizations of multi-kingdom gut microbiota in immune checkpoint inhibitor-treated hepatocellular carcinoma. J Immunother Cancer. (2024) 12(6):e008686. 10.1136/jitc-2023-008686 38844407 PMC11163665

[23] Tanaka Y Miki D Hayes CN Kawahara M Sueda S Emori T. A study on the association between treatment response to atezolizumab plus bevacizumab combination therapy for advanced hepatocellular carcinoma and gut microbiota. Microorganisms. (2026) 14(4):867. 10.3390/microorganisms14040867 42075264 PMC13119078

[24] Shen Y-C Lee P-C Kuo Y-L Wu W-K Chen C-C Lei C-H. An exploratory study for the association of gut microbiome with efficacy of immune checkpoint inhibitor in patients with hepatocellular carcinoma. J Hepatocell Carcinoma. (2021) 8:809. 10.2147/JHC.S315696 34336726 PMC8318216

[25] Zhao C-N Li S-S Yau T Chen W-Q Ji R Guan X-Y. Phocaeicola vulgatus induces immunotherapy resistance in hepatocellular carcinoma via reducing indoleacetic acid production. Cell Rep Med. (2025) 6(10):102370. 10.1016/j.xcrm.2025.102370 41005300 PMC12629828

[26] Fujii T Kuzuya T Kondo N Funasaka K Ohno E Hirooka Y. Altered intestinal Streptococcus anginosus and 5α-reductase gene levels in patients with hepatocellular carcinoma and elevated Bacteroides stercoris in atezolizumab/bevacizumab non-responders. J Med Microbiol. (2024) 73(9):001878. 10.1099/jmm.0.001878 39240069

[27] Barcena-Varela M Shang J Mogno I Lozano A Liebling I Mead KR. Bacteroidetes promote hepatocellular carcinoma progression and resistance to immunotherapy. *bioRxiv*. Published online December 12, 2025:2025.12.09.693238. 10.64898/2025.12.09.693238

[28] Shin HP Lee M. Navigating the therapeutic pathway and optimal first-line systemic therapy for hepatocellular carcinoma in the era of immune checkpoint inhibitors. Medicina. (2025) 61(12):2164. 10.3390/medicina61122164 41470166 PMC12735240

[29] Peddireddi RSS Kuchana SK Kode R Khammammettu S Koppanatham A Mattigiri S. Role of gut microbiome in oncogenesis and oncotherapies. Cancers. (2026) 18(1):99. 10.3390/cancers18010099 PMC12785007 41514614

[30] Routy B Le Chatelier E Derosa L Duong CPM Alou MT Daillère R. Gut microbiome influences efficacy of PD-1-based immunotherapy against epithelial tumors. Science. (2018) 359(6371):91–7. 10.1126/science.aan3706 29097494

[31] Cerreto M Maestri M Pallozzi M Cerrito L Stella L Ianiro G. Gut microbiota biomarkers in patients with hepatocellular carcinoma in the era of immune checkpoint inhibitors. Life. (2026) 16(4):641. 10.3390/life16040641 42073451 PMC13117110

[32] Bajaj JS Peña-Rodriguez M La Reau A Phillips W Fuchs M Davis BC. A longitudinal trans-kingdom gut microbial approach towards decompensation in outpatients with cirrhosis. Gut. (2023) 72(4):759–71. 10.1136/gutjnl-2022-328403 36343978 PMC9998342

[33] Feng M Chai Y Li J Wang Q Zhang D. A metagenome-wide association study of gut microbiota in hepatitis B virus-related cirrhosis in northwest China. Front Genet. (2025) 16:1619911. 10.3389/fgene.2025.1619911 40909440 PMC12404923

[34] Cassol I Ibañez M Bustamante JP. Key features and guidelines for the application of microbial alpha diversity metrics. Sci Rep. (2025) 15:622. 10.1038/s41598-024-77864-y 39753610 PMC11698868

[35] Huang J-H Wang J Chai X-Q Li Z-C Jiang Y-H Li J. The intratumoral bacterial metataxonomic signature of hepatocellular carcinoma. Microbiol Spectr. (2022) 10(5):e0098322. 10.1128/spectrum.00983-22 36173308 PMC9602924

[36] Wu XQ Ying F Chung KPS Leung CON Leung RWH So KKH. Intestinal Akkermansia muciniphila complements the efficacy of PD1 therapy in MAFLD-related hepatocellular carcinoma. Cell Rep Med. (2025) 6(1):101900. 10.1016/j.xcrm.2024.101900 39798567 PMC11866522

[37] Zhang H Wu J Liu Y Zeng Y Jiang Z Yan H. Identification reproducible microbiota biomarkers for the diagnosis of cirrhosis and hepatocellular carcinoma. AMB Express. (2023) 13:35. 10.1186/s13568-023-01539-6 36943499 PMC10030758

[38] Davar D Dzutsev AK McCulloch JA Rodrigues RR Chauvin J-M Morrison RM. Fecal microbiota transplant overcomes resistance to anti–PD-1 therapy in melanoma patients. Science. (2021) 371(6529):595–602. 10.1126/science.abf3363 33542131 PMC8097968

[39] Peters BA Wilson M Moran U Pavlick A Izsak A Wechter T. Relating the gut metagenome and metatranscriptome to immunotherapy responses in melanoma patients. Genome Med. (2019) 11:61. 10.1186/s13073-019-0672-4 31597568 PMC6785875

[40] Zheng C Lu F Chen B Yang J Yu H Wang D. Gut microbiome as a biomarker for predicting early recurrence of HBV-related hepatocellular carcinoma. Cancer Sci. (2023) 114(12):4717. 10.1111/cas.15983 37778742 PMC10728007

[41] Wang Y Yang Z Liu C Liu Y Bai Z Miao W. Gut microbial signatures of advanced hepatocellular carcinoma and their potential diagnostic value. Front Microbiol. (2026) 17:1760859. 10.3389/fmicb.2026.1760859 41704316 PMC12908660

[42] Peng Y-C Xu J-X You X-M Huang Y-Y Ma L Li L-Q. Specific gut microbiome signature predicts hepatitis B virus-related hepatocellular carcinoma patients with microvascular invasion. Ann Med. (2023) 55(2):2283160. 10.1080/07853890.2023.2283160 38112540 PMC10986448

[43] Chua H-H Chen Y-H Wu L-L Yang H-C Lin C-R Chen H-L. Antagonism between gut Ruminococcus gnavus and Akkermansia muciniphila modulates the progression of chronic hepatitis B. Cell Mol Gastroenterol Hepatol. (2024) 17(3):361–81. 10.1016/j.jcmgh.2023.12.003 38092311 PMC10821531

[44] Islam K Kongsomboonchoke P Chayanupatkul M Tumkosit M Chattranukulchai P Prombutara P. Gut dysbiosis and plasma trimethylamine oxide are associated with subclinical coronary atherosclerosis in obese patients with metabolic dysfunction-associated steatotic liver disease. Nutrients. (2025) 17(17):2759. 10.3390/nu17172759 40944149 PMC12430641

[45] Wu C Yang F Zhong H Hong J Lin H Zong M. Obesity-enriched gut microbe degrades myo-inositol and promotes lipid absorption. Cell Host Microbe. (2024) 32(8):1301–14.e9. 10.1016/j.chom.2024.06.012 38996548

[46] Che Z Xue W Zhao X Hu C Tian Y. Regulatory role and biomarker potential of gut microbiota metabolites in the progression of metabolic dysfunction-associated steatotic liver disease to hepatocellular carcinoma. Clin Transl Gastroenterol. (2025) 16(12):e00914. 10.14309/ctg.0000000000000914 40911047 PMC12727350

[47] Monti E Vianello C Leoni I Galvani G Lippolis A D’Amico F. Gut microbiome modulation in hepatocellular carcinoma: preventive role in NAFLD/NASH progression and potential applications in immunotherapy-based strategies. Cells. (2025) 14(2):84. 10.3390/cells14020084 39851512 PMC11764391

[48] Ma J Li J Jin C Yang J Zheng C Chen K. Association of gut microbiome and primary liver cancer: a two-sample Mendelian randomization and case-control study. Liver Int. (2023) 43(1):221–33. 10.1111/liv.15466 36300678

[49] Gopalakrishnan V Spencer CN Nezi L Reuben A Andrews MC Karpinets TV. Gut microbiome modulates response to anti–PD-1 immunotherapy in melanoma patients. Science. (2018) 359(6371):97–103. 10.1126/science.aan4236 29097493 PMC5827966

[50] Lan X Ma J Huang Z Xu Y Hu Y. Akkermansia muciniphila might improve anti-PD-1 therapy against HCC by changing host bile acid metabolism. J Gene Med. (2024) 26(1):e3639. 10.1002/jgm.3639 38058259

[51] Vandoni G D'Amico F Fabbrini M Mariani L Sieri S Casirati A. Gut microbiota, metabolome, and body composition signatures of response to therapy in patients with advanced melanoma. Int J Mol Sci. (2023) 24(14):11611. 10.3390/ijms241411611 37511376 PMC10380337

[52] Baruch EN Youngster I Ben-Betzalel G Ortenberg R Lahat A Katz L. Fecal microbiota transplant promotes response in immunotherapy-refractory melanoma patients. Science. (2021) 371(6529):602–9. 10.1126/science.abb5920 33303685

[53] Chen B-J Takeshita T Tajikara T Asakawa M Kageyama S Shibata Y. Butyrate as a potential driver of a dysbiotic shift of the tongue microbiota. mSphere. (2023) 8(1):e0049022. 10.1128/msphere.00490-22 36507724 PMC9942584

[54] Coutzac C Jouniaux J-M Paci A Schmidt J Mallardo D Seck A. Systemic short chain fatty acids limit antitumor effect of CTLA-4 blockade in hosts with cancer. Nat Commun. (2020) 11:2168. 10.1038/s41467-020-16079-x 32358520 PMC7195489

[55] Yang J Dai Y Li J. Gut microbiota–immunity cascade in hepatocellular carcinoma: mechanisms and therapeutic opportunities. Oncol Rev. (2026) 19:1687901. 10.3389/or.2025.1687901 41695569 PMC12894394

[56] Xu Y Tan X Yang Q Fang Z Chen W. Akkermansia muciniphila outer membrane protein regulates recruitment of CD8+ T cells in lung adenocarcinoma and through JAK–STAT signalling pathway. Microb Biotechnol. (2024) 17(7):e14522. 10.1111/1751-7915.14522 39016683 PMC11253302

[57] Muscolino P Granata B Omero F De Pasquale C Campana S Calabrò A. Potential predictive role of gut microbiota to immunotherapy in HCC patients: a brief review. Front Oncol. (2023) 13:1247614. 10.3389/fonc.2023.1247614 37692859 PMC10486017

[58] Ng KYY Tan SH Tan JJE Tay DSH Lee AWX Ang AJS. Impact of immune-related adverse events on efficacy of immune checkpoint inhibitors in patients with advanced hepatocellular carcinoma. Liver Cancer. (2022) 11(1):9–21. 10.1159/000518619 35222504 PMC8820151

[59] Hu M Lin X Sun T Shao X Huang X Du W. Gut microbiome for predicting immune checkpoint blockade-associated adverse events. Genome Med. (2024) 16:16. 10.1186/s13073-024-01285-9 38243343 PMC10799412

[60] Hashem HR Yehia T Azab M Abdellah A Amin IA Salah M. Gut microbiome dysbiosis in hepatocellular carcinoma patients with persistent HCV viremia versus viral clearance: a cross-sectional study. Gut Pathog. (2025) 17:88. 10.1186/s13099-025-00761-w 41239524 PMC12619186

[61] Satapathy SK Banerjee P Pierre JF Higgins D Dutta S Heda R. Characterization of gut microbiome in liver transplant recipients with nonalcoholic steatohepatitis. Transplant Direct. (2020) 6(12):e625. 10.1097/TXD.0000000000001033 33204823 PMC7665248

[62] Vallianou N Christodoulatos GS Karampela I Tsilingiris D Magkos F Stratigou T. Understanding the role of the gut microbiome and microbial metabolites in non-alcoholic fatty liver disease: current evidence and perspectives. Biomolecules. (2022) 12(1):56. 10.3390/biom12010056 PMC8774162 35053205

[63] Cavallari JF Pokrajac NT Zlitni S Foley KP Henriksbo BD Schertzer JD. NOD2 In hepatocytes engages a liver-gut axis to protect against steatosis, fibrosis, and gut dysbiosis during fatty liver disease in mice. Am J Physiol Endocrinol Metab. (2020) 319(2):E305–14. 10.1152/ajpendo.00181.2020 32516028

[64] Gao Y Liu W Ma X Xue M Li Y Bi S. The role of intestinal microbiota and its metabolites in the occurrence and intervention of obesity. Front Microbiol. (2025) 16:1559178. 10.3389/fmicb.2025.1559178 40384781 PMC12081357

