# Supplementary material for: Gut microbiota composition in responders vs. non-responders with hepatocellular carcinoma under ICI therapy: a systematic review and meta-analysis
Source: Front Med (Lausanne). 2026 Sep 9;13:1928103. doi: 10.3389/fmed.2026.1928103 (PMC13597227; doi:10.3389/fmed.2026.1928103)
Supplement: Supplementary file 1 [file Table1.docx]

Supplementary Table 1. Literature search strategy.

1.Pubmed

| Search number | Query | Results |
| --- | --- | --- |
| #1 | "Gastrointestinal Microbiome"[Mesh] | 68,573 |
| #2 | ((((((((((((((((((((((((((((((((((((((Gastrointestinal Microbiome[Title/Abstract]) OR (Gastrointestinal Microbiomes[Title/Abstract])) OR (Microbiome, Gastrointestinal[Title/Abstract])) OR (Gut Microbiome[Title/Abstract])) OR (Gut Microbiomes[Title/Abstract])) OR (Microbiome, Gut[Title/Abstract])) OR (Gut Microflora[Title/Abstract])) OR (Microflora, Gut[Title/Abstract])) OR (Gut Microbiota[Title/Abstract])) OR (Gut Microbiotas[Title/Abstract])) OR (Microbiota, Gut[Title/Abstract])) OR (Gastrointestinal Flora[Title/Abstract])) OR (Flora, Gastrointestinal[Title/Abstract])) OR (Gut Flora[Title/Abstract])) OR (Flora, Gut[Title/Abstract])) OR (Gastrointestinal Microbiota[Title/Abstract])) OR (Gastrointestinal Microbiotas[Title/Abstract])) OR (Microbiota, Gastrointestinal[Title/Abstract])) OR (Gastrointestinal Microbial Community[Title/Abstract])) OR (Gastrointestinal Microbial Communities[Title/Abstract])) OR (Microbial Community, Gastrointestinal[Title/Abstract])) OR (Gastrointestinal Microflora[Title/Abstract])) OR (Microflora, Gastrointestinal[Title/Abstract])) OR (Gastric Microbiome[Title/Abstract])) OR (Gastric Microbiomes[Title/Abstract])) OR (Microbiome, Gastric[Title/Abstract])) OR (Intestinal Microbiome[Title/Abstract])) OR (Intestinal Microbiomes[Title/Abstract])) OR (Microbiome, Intestinal[Title/Abstract])) OR (Intestinal Microbiota[Title/Abstract])) OR (Intestinal Microbiotas[Title/Abstract])) OR (Microbiota, Intestinal[Title/Abstract])) OR (Intestinal Microflora[Title/Abstract])) OR (Microflora, Intestinal[Title/Abstract])) OR (Intestinal Flora[Title/Abstract])) OR (Flora, Intestinal[Title/Abstract])) OR (Enteric Bacteria[Title/Abstract])) OR (Bacteria, Enteric[Title/Abstract])) OR ("Gastrointestinal Microbiome"[Mesh]) | 139992 |
| #3 | Hepatocellular carcinoma[MeSH] | 119888 |
| #4 | ((((((((((Hepatocellular carcinoma[Title/Abstract]) OR (HCC[Title/Abstract])) OR (Liver cell carcinoma[Title/Abstract])) OR (Hepatoma[Title/Abstract])) OR (Malignant hepatoma[Title/Abstract])) OR (Hepatocarcinoma[Title/Abstract])) OR (Primary hepatocellular carcinoma[Title/Abstract])) OR (Primary liver cell carcinoma[Title/Abstract])) OR (Liver cell cancer[Title/Abstract])) OR (Hepatocellular cancer[Title/Abstract])) OR (Carcinoma hepatocellular[Title/Abstract]) | 181845 |
| #5 | Immune checkpoint inhibitors[MeSH] | 19657 |
| #6 | ((((((((((((Immune checkpoint inhibitors[Title/Abstract]) OR (Immune checkpoint inhibitor[Title/Abstract])) OR (Checkpoint inhibitors[Title/Abstract])) OR (Checkpoint inhibitor[Title/Abstract])) OR (ICIs[Title/Abstract])) OR (Anti-PD-1/PD-L1 therapy[Title/Abstract])) OR (Anti-CTLA-4 therapy[Title/Abstract])) OR (PD-1 inhibitor[Title/Abstract])) OR (PD-L1 inhibitors[Title/Abstract])) OR (CTLA-4 inhibitors[Title/Abstract])) OR (Immune checkpoint blockade[Title/Abstract])) OR (Checkpoint blockade immunotherapy[Title/Abstract])) OR (Immune checkpoint blocking agents[Title/Abstract]) | 62111 |
| #7 | ((#2) AND (#4)) AND (#6) | 96 |
| #8 | ((((((((Atezolizumab[Title/Abstract]) OR (Nivolumab[Title/Abstract])) OR (Pembrolizumab[Title/Abstract])) OR (Tremelimumab[Title/Abstract])) OR (Durvalumab[Title/Abstract])) OR (Zimberelimab[Title/Abstract])) OR (Tislelizumab[Title/Abstract])) OR (Camrelizumab[Title/Abstract])) OR (Toripalimab[Title/Abstract]) | 29570 |
| #9 | #7 AND #8 | 17 |

2.Cochrane

| Search number | Query | Results |
| --- | --- | --- |
| #1 | MeSH descriptor: Gastrointestinal Microbiome  Explode all trees | 2282 |
| #2 | (Gastrointestinal Microbiome):ti,ab,kw OR (Gastrointestinal Microbiomes):ti,ab,kw OR (Microbiome, Gastrointestinal):ti,ab,kw OR (Gut Microbiome):ti,ab,kw OR (Gut Microbiomes):ti,ab,kw | 5630 |
| #3 | (Microbiome, Gut):ti,ab,kw OR (Gut Microflora):ti,ab,kw OR (Microflora, Gut):ti,ab,kw OR (Gut Microbiota):ti,ab,kw OR (Gut Microbiotas):ti,ab,kw | 9196 |
| #4 | (Microbiome, Gastric):ti,ab,kw OR (Intestinal Microbiome):ti,ab,kw OR (Intestinal Microbiomes):ti,ab,kw OR (Microbiome, Intestinal):ti,ab,kw OR (Intestinal Microbiota):ti,ab,kw | 3955 |
| #5 | (Intestinal Microbiotas):ti,ab,kw OR (Microbiota, Intestinal):ti,ab,kw OR (Intestinal Microflora):ti,ab,kw OR (Microflora, Intestinal):ti,ab,kw OR (Intestinal Flora):ti,ab,kw | 5112 |
| #6 | (Flora, Intestinal):ti,ab,kw OR (Enteric Bacteria):ti,ab,kw OR (Bacteria, Enteric):ti,ab,kw | 3049 |
| #7 | #2 OR #3 OR #4 OR #5 OR #6 | 13172 |
| #8 | MeSH descriptor: Carcinoma, Hepatocellular  Explode all trees | 2925 |
| #9 | (Hepatocellular carcinoma):ti,ab,kw OR (HCC):ti,ab,kw OR (Liver cell carcinoma):ti,ab,kw OR (Hepatoma):ti,ab,kw OR (Malignant hepatoma):ti,ab,kw | 9458 |
| #10 | (Hepatocarcinoma):ti,ab,kw OR (Primary liver carcinoma):ti,ab,kw OR (Primary hepatocellular carcinoma):ti,ab,kw OR (Liver cell cancer):ti,ab,kw OR (Hepatocellular cancer):ti,ab,kw | 9030 |
| #11 | #8 OR #9 OR #10 | 12355 |
| #12 | MeSH descriptor: Immune Checkpoint Inhibitors  Explode all trees | 487 |
| #13 | (Immune checkpoint inhibitor):ti,ab,kw OR (Checkpoint inhibitor):ti,ab,kw OR (ICIs):ti,ab,kw OR (Anti-PD-1/PD-L1 therapy):ti,ab,kw OR (Anti-CTLA-4 therapy):ti,ab,kw | 2170 |
| #14 | (PD-1 inhibitors):ti,ab,kw OR (CTLA-4 inhibitors):ti,ab,kw OR (Immune checkpoint blockade):ti,ab,kw OR (Checkpoint blockade immunotherapy):ti,ab,kw OR (Immune checkpoint blocking agents):ti,ab,kw | 1945 |
| #15 | #13 OR #14 | 3967 |
| #16 | #7 AND #11 AND #15 | 6 |
| #17 | (Atezolizumab):ti,ab,kw OR (Nivolumab):ti,ab,kw OR (Pembrolizumab):ti,ab,kw OR (Tremelimumab):ti,ab,kw OR (Durvalumab):ti,ab,kw | 11386 |
| #18 | (Zimberelimab):ti,ab,kw OR (Tislelizumab):ti,ab,kw OR (Camrelizumab):ti,ab,kw OR (Toripalimab):ti,ab,kw | 1296 |
| #19 | #17 OR #18 | 12608 |
| #20 | #16 AND #19 | 3 |

3.Embase

| Search number | Query | Results |
| --- | --- | --- |
| #1 | ('intestine flora')/exp | 159,647 |
| #2 | 'intestine flora':ti,ab,kw OR 'alimentary canal flora':ti,ab,kw OR 'alimentary tract flora':ti,ab,kw OR 'bowel flora':ti,ab,kw OR 'bowel microbiota':ti,ab,kw OR 'digestive canal flora':ti,ab,kw OR 'digestive tract flora':ti,ab,kw OR 'enteric flora':ti,ab,kw OR 'enteric microbiota':ti,ab,kw OR 'flora, intestine':ti,ab,kw OR 'gastro intestinal flora':ti,ab,kw OR 'gastrointestinal canal flora':ti,ab,kw OR 'gastrointestinal flora':ti,ab,kw OR 'gastrointestinal microbiome':ti,ab,kw OR 'gastrointestinal microbiota':ti,ab,kw OR 'gastrointestinal tract flora':ti,ab,kw OR 'gastrointestine flora':ti,ab,kw OR 'gastrointestine tract flora':ti,ab,kw OR 'gut bacteria':ti,ab,kw OR 'gut microbiota':ti,ab,kw OR 'intestinal bacteria':ti,ab,kw OR 'intestinal bacterial flora':ti,ab,kw OR 'intestinal bacterium':ti,ab,kw OR 'intestinal canal flora':ti,ab,kw OR 'intestinal flora':ti,ab,kw OR 'intestinal microbe':ti,ab,kw OR 'intestinal microbes':ti,ab,kw OR 'intestinal microbiota':ti,ab,kw OR 'intestinal microflora':ti,ab,kw OR 'intestinal microorganism':ti,ab,kw OR 'intestinal tract flora':ti,ab,kw OR 'intestine bacteria':ti,ab,kw OR 'intestine bacteria change':ti,ab,kw OR 'intestine bacterial flora':ti,ab,kw OR 'intestine bacterium':ti,ab,kw OR 'intestine microbial flora':ti,ab,kw OR 'intestine microflora':ti,ab,kw | 129,766 |
| #3 | #1 OR #2 | 182,642 |
| #4 | ('Hepatocellular carcinoma')/exp | 260,936 |
| #5 | ('Hepatocellular carcinoma'):ti,ab,kw OR ((HCC):ti,ab,kw) OR (('Liver cell carcinoma'):ti,ab,kw) OR ((Hepatoma):ti,ab,kw) OR (('Malignant hepatoma'):ti,ab,kw) OR ((Hepatocarcinoma):ti,ab,kw) OR (('Primary liver carcinoma'):ti,ab,kw) OR (('Primary hepatocellular carcinoma'):ti,ab,kw) OR (('Liver cell cancer'):ti,ab,kw) OR (('Hepatocellular cancer'):ti,ab,kw) | 267,986 |
| #6 | #4 OR #5 | 323,390 |
| #7 | ('Immune checkpoint inhibitor')/exp | 59,141 |
| #8 | ('Immune checkpoint inhibitor'):ti,ab,kw OR (('Checkpoint inhibitor'):ti,ab,kw) OR ((ICIs):ti,ab,kw) OR (('Anti-PD-1/PD-L1 therapy'):ti,ab,kw) OR (('Anti-CTLA-4 therapy'):ti,ab,kw) OR (('PD-1 inhibitors'):ti,ab,kw) OR (('CTLA-4 inhibitors'):ti,ab,kw) OR (('Immune checkpoint blockade'):ti,ab,kw) OR (('Checkpoint blockade immunotherapy'):ti,ab,kw) OR (('Immune checkpoint blocking agents'):ti,ab,kw) | 67,353 |
| #9 | (Atezolizumab):ti,ab,kw OR ((Nivolumab):ti,ab,kw) OR ((Pembrolizumab):ti,ab,kw) OR ((Tremelimumab):ti,ab,kw) OR ((Durvalumab):ti,ab,kw) OR ((Zimberelimab):ti,ab,kw) OR ((Tislelizumab):ti,ab,kw) OR ((Camrelizumab):ti,ab,kw) OR ((Toripalimab):ti,ab,kw) | 74,652 |
| #10 | #7 OR #8 OR #9 | 148,448 |
| #11 | #3 AND #6 AND #10 | 234 |

4.Web of science

| Search number | Query | Results |
| --- | --- | --- |
| #1 | ((((((((((((((((((TS=(Gastrointestinal Microbiome)) OR TS=(Gastrointestinal Microbiomes)) OR TS=( Gut Microbiome)) OR TS=(Gut Microflora)) OR TS=(Microflora)) OR TS=(Gut Microbiota)) OR TS=(Gastrointestinal Flora)) OR TS=(Gastrointestinal )) OR TS=(Gut Flora)) OR TS=(Flora)) OR TS=(Gastrointestinal Microbiota)) OR TS=(Gastrointestinal Microbial Community)) OR TS=(Gastrointestinal Microflora)) OR TS=(Microflora, Gastrointestinal)) OR TS=(Gastric Microbiome )) OR TS=(Intestinal Microbiome)) OR TS=(Intestinal Microflora)) OR TS=(Intestinal Flora)) OR TS=(Enteric Bacteria) | 1,354,235 |
| #2 | (((((((((TS=(Hepatocellular carcinoma)) OR TS=(HCC)) OR TS=(Liver cell carcinoma)) OR TS=(Hepatoma)) OR TS=(Malignant hepatoma)) OR TS=(Hepatocarcinoma)) OR TS=(Primary liver carcinoma)) OR TS=(Primary hepatocellular carcinoma)) OR TS=(Liver cell cancer)) OR TS=(Hepatocellular cancer) | 544,488 |
| #3 | (((((((((TS=(Immune checkpoint inhibitor)) OR TS=(Checkpoint inhibitor)) OR TS=(ICIs)) OR TS=(Anti-PD-1/PD-L1 therapy)) OR TS=(Anti-CTLA-4 therapy)) OR TS=(PD-1 inhibitors)) OR TS=(CTLA-4 inhibitors)) OR TS=(Immune checkpoint blockade)) OR TS=(Checkpoint blockade immunotherapy)) OR TS=(Immune checkpoint blocking agents) | 137,989 |
| #4 | ((((((((TS=(Atezolizumab)) OR TS=(Nivolumab)) OR TS=(Pembrolizumab)) OR TS=(Tremelimumab)) OR TS=(Durvalumab)) OR TS=(Zimberelimab)) OR TS=(Tislelizumab)) OR TS=(Camrelizumab)) OR TS=(Toripalimab) | 73,958 |
| #5 | #1 AND #2 AND #3 AND #4  https://www.webofscience.com/wos/alldb/summary/747e1c2b-d8f6-479d-9cec-9112828ab254-01b6d3a7e9/relevance/1 | 327 |

Supplementary Table 2. Quality assessment of the included cohort studies using the Newcastle-Ottawa Scale.

| Author | Year | Selection | | | | Comparability | Outcome | | | score |
| --- | --- | --- | --- | --- | --- | --- | --- | --- | --- | --- |
|  |  | Representativeness of the exposed cohort | Selection of the non exposed cohort | Ascertainment of exposure | Demonstration that outcome of interest was not present at start of study | Comparability of cohorts on the basis of the design or analysis | Assessment of outcome | Was follow-up long enough for outcomes to occur | Adequacy of follow up of cohorts |  |
| Lee | 2022 | 1 | 1 | 1 | 1 | 1 | 1 | 0 | 0 | 6 |
| Inukai | 2024 | 1 | 1 | 1 | 1 | 1 | 1 | 0 | 1 | 7 |
| Chung | 2021 | 1 | 1 | 1 | 1 | 1 | 1 | 1 | 0 | 7 |
| Xin | 2024 | 1 | 1 | 1 | 1 | 1 | 1 | 1 | 0 | 7 |
| Mao | 2021 | 1 | 1 | 1 | 1 | 1 | 1 | 1 | 0 | 7 |
| Ponziani | 2022 | 1 | 1 | 1 | 1 | 1 | 1 | 0 | 0 | 6 |
| Zheng | 2019 | 1 | 1 | 1 | 1 | 1 | 1 | 0 | 0 | 6 |
| Zhu | 2024 | 1 | 1 | 1 | 1 | 1 | 1 | 1 | 0 | 7 |
| Tanaka | 2026 | 1 | 1 | 1 | 1 | 1 | 1 | 1 | 1 | 8 |
| Shen | 2021 | 1 | 1 | 1 | 1 | 1 | 1 | 1 | 1 | 8 |
| Zhao | 2025 | 1 | 1 | 1 | 1 | 2 | 1 | 1 | 1 | 9 |
| Fujii | 2024 | 1 | 1 | 1 | 1 | 2 | 1 | 1 | 0 | 8 |
| Varela | 2025 | 1 | 1 | 1 | 1 | 2 | 1 | 1 | 0 | 8 |

Supplementary Figure 1. A meta-analysis of the reported microbial taxa at Bacteroidetes and Proteobacteria after excluding two outlier studies.

Supplementary Figure 2. A meta-analysis of the reported microbial taxa at Family level. Supplementary Figure 3. A meta-analysis of the reported microbial taxa at Species level.

Supplementary Figure 4. Changes of relative abundance of 24 phyla included in analysis.

Supplementary Figure 5. Changes of relative abundance of 26 genara included in analysis.

Supplementary Figure 6. Changes of relative abundance of 15 families included in analysis.

Supplementary Table 3. Summary of survival associations of characteristic gut bacterial taxa for overall survival (OS).

| Study | OS | Taxa | Phylum | HR | CI | P |
| --- | --- | --- | --- | --- | --- | --- |
| Lee2022 | OS | Prevotella 9 | Bacteroidota | 2.04 | 0.93-4.49 | 0.076 |
| Lee2022 | OS | Lachnoclostridium | Firmicutota | 0.42 | 0.17-1.03 | 0.058 |
| Inukai2024 | OS | Bacteroides stercoris/ Parabacteroides merdae | Bacteroidota | 0.34 | 0.11-1.00 | 0.049 |
| Xin2024 | OS | Collinsella | Actinobacteriota | 0.31 | 0.10–0.93 | 0.02 |
| Xin2024 | OS | Ruminococcus.AM42_11 | Firmicutota | 0.29 | 0.11–0.77 | 0.007 |
| Xin2024 | OS | Bacteroides.AF20_13LB | Bacteroidota | 8.16 | 3.04–21.88 | <0.001 |
| Xin2024 | OS | Veillonella.atypica | Firmicutota | 3.92 | 1.53–10.02 | 0.006 |
| Xin2024 | OS | Veillonella.AF13_2 | Firmicutota | 5.24 | 1.86–14.75 | 0.004 |
| Xin2024 | OS | Ruminococcus.AF25_28AC | Firmicutota | 0.16 | 0.04–0.69 | 0.002 |
| Mao2021 | OS | Erysipelotrichaceae bacterium-GAM147 | Firmicutota | 0.15 | 0.02-1.17 | 0.041 |
| Mao2021 | OS | Veillonellaceae family | Firmicutota | 5.24 | 1.05-26.20 | 0.03 |
| Mao2021 | OS | Bacteroidales | Bacteroidota | 1.52 | 0.72-3.19 | 0.26 |
| Mao2021 | OS | Bacteroidales zoogleoformans | Bacteroidota | 1.42 | 0.62-3.23 | 1.416 |
| Zhu2024 | OS | s_Actinomyces_sp_ICM47 | Actinobacteriota | 1.98 | 1.00-3.90 | 0.048 |
| Zhu2024 | OS | s_Senegalimassilia_anaerobia | Firmicutota | 1.03 | 0.44-2.44 | 0.943 |
| Zhao2024 | OS | P. vulgatus | Bacteroidota | 1.48 | 0.91-2.41 | 0.114 |
| Shen2021 | OS | Bifidobacterium | Actinobacteriota | 0.50 | 0.20-1.27 | 0.143 |
| Shen2021 | OS | Coprococcus | Firmicutota | 0.95 | 0.38-2.39 | 0.917 |
| Shen2021 | OS | Acidaminococcus | Firmicutota | 0.75 | 0.30-1.86 | 0.529 |

Supplementary Table 4. Summary of survival associations of characteristic gut bacterial taxa for progression-free survival (PFS).

| Study | PFS | Taxa | Phylum | HR | CI | P |
| --- | --- | --- | --- | --- | --- | --- |
| Lee2022 | PFS | Prevotella 9 | Bacteroidota | 2.72 | 1.25-5.93 | 0.012 |
| Lee2022 | PFS | Lachnoclostridium | Firmicutota | 0.502 | 0.21-1.19 | 0.117 |
| Inukai2024 | PFS | Bacteroides stercoris/ Parabacteroides merdae | Bacteroidota | 0.54 | 0.19-1.56 | 0.26 |
| Xin2024 | PFS | Collinsella | Actinobacteriota | 0.29 | 0.13–0.68 | 0.002 |
| Xin2024 | PFS | Ruminococcus.AM42_11 | Firmicutota | 0.51 | 0.26–1.01 | 0.048 |
| Xin2024 | PFS | Bacteroides.AF20_13LB | Bacteroidota | 11.18 | 3.77–33.14 | <0.001 |
| Xin2024 | PFS | Veillonella.atypica | Firmicutota | 2.46 | 1.19–5.06 | 0.02 |
| Xin2024 | PFS | Veillonella.AF13_2 | Firmicutota | 2.53 | 1.08–5.94 | 0.051 |
| Xin2024 | PFS | Ruminococcus.AF25_28AC | Firmicutota | 0.15 | 0.06–0.38 | <0.001 |
| Mao2021 | PFS | Erysipelotrichaceae bacterium-GAM147 | Firmicutota | 0.31 | 0.11-0.88 | 0.021 |
| Mao2021 | PFS | Veillonellaceae family | Firmicutota | 4.42 | 1.41-13.88 | 0.005 |
| Mao2021 | PFS | Bacteroidales | Bacteroidota | 1.372 | 0.80-2.36 | 0.25 |
| Mao2021 | PFS | Bacteroidales zoogleoformans | Bacteroidota | 0.53 | 0.29-0.98 | 0.043 |
| Mao2021 | PFS | Ruminococcus callidus | Firmicutota | 0.29 | 0.11-0.75 | 0.006 |
| Zhu2024 | PFS | s_Actinomyces_sp_ICM47 | Actinobacteriota | 1.49 | 0.77-2.89 | 0.241 |
| Zhu2024 | PFS | s_Senegalimassilia_anaerobia | Firmicutota | 2.35 | 0.98-5.63 | 0.056 |
| Zhao2024 | PFS | P. vulgatus | Bacteroidota | 1.47 | 0.90-2.40 | 0.123 |
| Tanaka2026 | PFS | Acidaminococcus | Firmicutota | 0.437 | 0.15-1.31 | 0.139 |
| Tanaka2026 | PFS | Lachnoclostridium | Firmicutota | 2.41 | 1.04-5.57 | 0.039 |
| Tanaka2026 | PFS | Flavonifractor | Firmicutota | 1.34 | 0.59-3.04 | 0.478 |
| Tanaka2026 | PFS | Megamonas | Firmicutota | 0.36 | 0.12-1.07 | 0.066 |

Supplementary Figure 7. Changes of part of KEGG included in analysis.

| Study | Group | Metabolism of the fecal microbiota | Trend | Associated microbiota |
| --- | --- | --- | --- | --- |
| Lee et al[18] | R | primary and secondary bile acids; isohyodeoxycholic acid; nutriacholic acid | ↑ | NA |
|  |  | oxypurinol, inosine and D-glucuronic acid | ↓ | NA |
|  |  | UDCA, UCA and MDCA | ↑ | *Lachnoclostridium* and *Ruminococcus gnavus* (+) |
|  | NR | secondary bile | ↑ | *Prevotella 9* (−) |
| Mao et al[19] | R | aurachin metabolism, fatty acid biosynthesis, acarbose biosynthesis, and polyketide sugar unit biosynthesis. | ↑ | NA |
|  |  | bacterial reproduction-related pathways (e.g., UDP/UMP biosynthesis) | ↑ | NA |
|  | NR | amino acid metabolism pathways (e.g., arginine biosynthesis) | ↑ | NA |
| Zheng et al[20] | R | the cellobiose-transport system (KO02010) | ↑ | *Bacteroides. dentium*(+) |
|  |  | pectin lyase (K01732, pectin metabolism) | ↑ | *Akkermansia. muciniphila*(+) |
|  |  | methanogenesis (KO00680) | ↑ | *Ruminococcus. obeum* and four *Lactobacillus* species (+) |
|  |  | including sulfate reduction, carbon fixation, carotenoid biosynthesis, and unsaturated fatty acid metabolism | ↑ | key responder-enriched species (+) |
| Zhu et al[17] | R | KO00750 | ↑ | NA |
|  | NR | KO00250 and KO00910 | ↑ | NA |
| Zhao et al[21] | NR | iorA (K00179) and iorB (K00180) | ↑ | *Phocaeicola. vulgatus*(+) |
|  |  | ipdC (K04103) | ↓ | *Phocaeicola. vulgatus*(+) |

Supplementary Table 5. Summary of differences in the metabolism of the fecal microbiota.

(+): positive correlation; (−): negative correlation; NA, Not announced.

Supplementary Table 6. Summary of the clinical factors of the fecal microbiota.

| Study | Group | Clinical factors | Associated microbiota |
| --- | --- | --- | --- |
| Lee et al[18] | NA | overall survival | enriched *Lachnoclostridium* and depleted *Prevotella 9*(+) |
| Mao et al[19] | NA | severe diarrhea | *Prevotellamassilia timonensis*(+) |
|  |  | Mild diarrhea | *Firmicutes* phylum(+) |
|  |  | large tumors (≥5 cm). | *Eubacterium hallii*(+) |
|  | NR | elevated bile acid, bilirubin and impaired liver function | *Veillonellaceae, Dialister sp. Marseille-P5638, Clostridiaceae, Klebsiella and Citrobacter freundii*(+) |
|  | R | tumor number and stage | *Faecalibacterium prausnitzii*(−) |
| Zhu et al[17] | NA | total bilirubin and bile acids | *Clostridium scindens and Clostridia bacterium UC5_1_1D1*(+) |
|  |  | aspartate aminotransferase, gamma glutamyl transferase, alanine aminotransferase, tumor size, and vascular invasion | *s_Candidatus_Avimonas_narfia*(+) |
| Shen et al[23]. | NA | no antibiotic use in the 4 weeks prior to immunotherapy and absence of prior systemic treatment | *Acidaminococcus* and *Coprococcus Acidaminococcus and Coprococcus*(+) |

(+): positive correlation; (−): negative correlation; NA, Not announced.

Supplementary Table S7. Baseline heterogeneity of the included studies.

| **Author** | **country** | **Etiology** | **Stage** | **Combined therapy** | **Exclusion of influencing factors (antibiotics, yogurt, probiotics, proton pump inhibitors, etc.)** | **Radiological criteria** |
| --- | --- | --- | --- | --- | --- | --- |
| Inukai2024 | Japan | Hepatitis (51.3%); Alcohol (32.4%) | BCLC stage B/C (91.8%) | NA | NO | RECIST |
| Chung2021 | Korea | HBV (75%) | BCLC stage C | NA | NA | RECIST |
| Lee2022 | China | HBV (63.4%) | BCLC stage B/C | TACE/RFA+Sorafenib | Yes | RECIST |
| Mao2021 | China | Hepatitis (50%) | III-IV (90%) | NA | Yes | RECIST |
| Ponziani2022 | Italy | Hepatitis (45.4%), NAFLD (27%), Alcohol (27%) | advanced-stage | NA | Yes | mRECIST |
| Xin2024 | China | HBV (95.6%) | BCLC stage B/C | Lenvatinib+cTACE | Yes | mRECIST |
| Zheng2019 | China | NA | BCLC Stage C | NA | Yes | RECIST |
| Zhu2024 | China | Hepatitis (91.8%) | TNM III-IV (91.8%) | Lenvatinib | Yes | RECIST |
| Tanaka2026 | Japan | Hepatitis (65.5%) | BCLC stage B/C (87%) | NA | NO | mRECIST |
| Shen2021 | China | HBV (69.4%) | advanced-stage | Lenvatinib | NO | RECIST |
| Zhao2025 | China | HBV (81.5%) | BCLC stage B/C | TACE/RFA+targeted therapy | NA | mRECIST |
| Fujii2024 | Japan | NA | BCLC stage B/C | NA | NA | RECIST |
| Varela2025 | USA | NA | advanced-stage | Lenvatinib | NA | RECIST |

Table Notes: HBV, hepatitis B virus; BCLC, Barcelona Clinic Liver Cancer; TNM, tumor node metastasis classification; TACE, Transarterial chemoembolization; RFA, Radio Frequency Ablation; RECIST, Response Evaluation Criteria in Solid Tumors; NA, Not announced.

Supplementary Figure 8. Funnel plot on pooled SMD for assessing publication bias.

Supplementary Figure 9. Trim-and-fill funnel plot on pooled SMD for assessing publication bias.

 The y‑axis represents the standard error of SMD, and the x‑axis represents SMD. Solid circles denote observed original studies; open circle represents the hypothetically missing study imputed by trim‑and‑fill algorithm. Dashed lines show pooled effect and funnel boundaries.
